# Supplementary material for: Range extender mediates long-distance enhancer activity
Source: Nature. 2025 Jul 2;643(8072):830–8. doi: 10.1038/s41586-025-09221-6 (PMC12267059; doi:10.1038/s41586-025-09221-6)
Supplement: Supplementary file 1 — Supplementary Fig. 1, Supplementary Note 1 and legends for Supplementary Tables 1–6. [file 41586_2025_9221_MOESM1_ESM.pdf]

nature portfolio

https://doi.org/10.1038/s41586-025-09221-6

Supplementary information

Range extender mediates long-distance enhancer activity

In the format provided by the authors and unedited

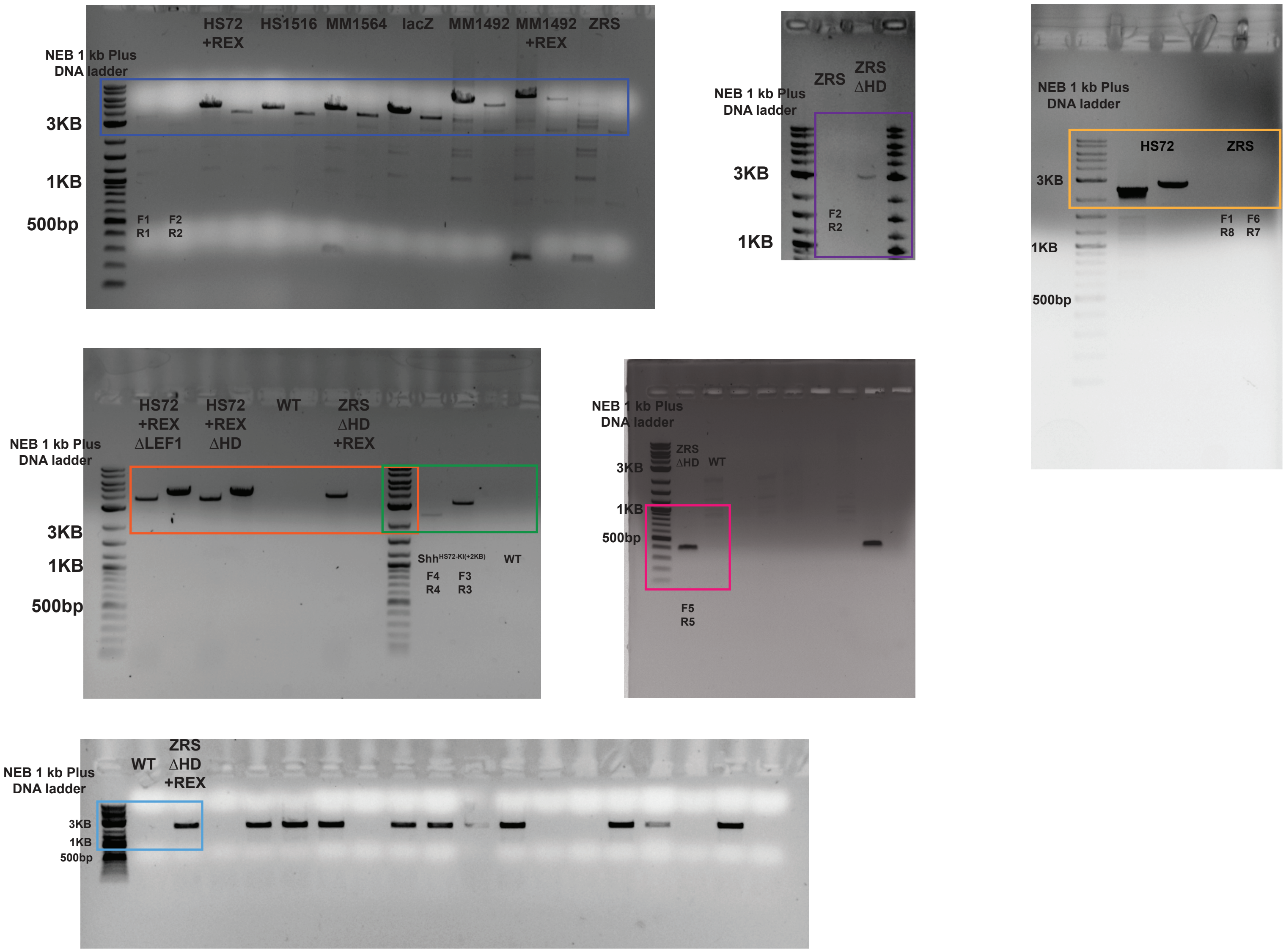

Supplementary Data Figure 1

Supplementary Note 1

Vector Sequences

1. ZRS-MHA-PCR4-HS72 (Targeting Vector)

PCR4-TOPO backbone
ZRS Homology Arms
Enhancer (REX Element)

gctaaattcaattcgccctatagtgagtcgtattacaattcactggccgtcgttttacaacgtcgtgactgggaaaaccctggcgttacccaacttaatcgccttgcagcacatccccctttcgccagctggcgtaatagcgaagaggcccgcaccgatcgcccttccca
acagttgcgcagcctatacgtacggcagtttaaggtttacacctataaaagagagagccgttatcgtctgtttgtggatgtacagagtgatattattgacacgccggggcgacggatggtgatccccctggccagtgcacgtctgctgtcagataaagtctcccgtgaac
tttacccggtggtgcatatcggggatgaaagctggcgcatgatgaccaccgatatggccagtgtgccggtctccgttatcggggaagaagtggctgatctcagccaccgcgaaaatgacatcaaaaacgccattaacctgatgttctggggaatataaatgtcaggc
atgagattatcaaaaaggatcttcacctagatccttttcacgtagaaagccagtccgcagaaacggtgctgaccccggatgaatgtcagctactgggctatctggacaagggaaaacgcaagcgcaaagagaaagcaggtagcttgcagtgggcttacatggcg
atagctagactgggcggttttatggacagcaagcgaaccggaattgccagctggggcgccctctggtaaggttgggaagccctgcaaagtaaactggatggctttctcgccgccaaggatctgatggcgcaggggatcaagctctgatcaagagacaggatgag
gatcgtttcgcatgattgaacaagatggattgcacgcaggttctccggccgcttgggtggagaggctattcggctatgactgggcacaacagacaatcggctgctctgatgccgccgtgttccggctgtcagcgcaggggcgcccggttctttttgtcaagaccgacct
gtccggtgccctgaatgaactgcaagacgaggcagcgcggctatcgtggctggccacgacgggcgttccttgcgcagctgtgctcgacgttgtcactgaagcgggaagggactggctgctattgggcgaagtgccggggcaggatctcctgtcatctcaccttgctc
ctgccgagaaagtatccatcatggctgatgcaatgcggcggctgcatacgcttgatccggctacctgcccattcgaccaccaagcgaaacatcgcatcgagcgagcacgtactcggatggaagccggtcttgtcgatcaggatgatctggacgaagagcatcag
gggctcgcgccagccgaactgttcgccaggctcaaggcgagcatgcccgacggcgaggatctcgtcgtgacccatggcgatgcctgcttgccgaatatcatggtggaaaatggccgcttttctggattcatcgactgtggccggctgggtgtggcggaccgctatca
ggacatagcgttggctacccgtgatattgctgaagagcttggcggcgaatgggctgaccgcttcctcgtgctttacggtatcgccgctcccgattcgcagcgcatcgccttctatcgccttcttgacgagttcttctgaattattaacgcttacaatttcctgatgcggtattttct
ccttacgcatctgtgcggtatttcacaccgcatacaggtggcacttttcggggaaatgtgcgcggaacccctatttgtttatttttctaaatacattcaaatatgtatccgctcatgagacaataaccctgataaatgcttcaataatattgaaaaaggaagagtatgagtattc
aacatttccgtgtcgcccttattcccttttttgcggcattttgccttcctgtttttgctcacccagaaacgctggtgaaagtaaaagatgctgaagatcagttgggtgcacgagtgggttacatcgaactggatctcaacagcggtaagatccttgagagttttcgccccgaag
aacgttttccaatgatgagcacttttaaagttctgctatgtggcgcggtattatcccgtattgacgccgggcaagagcaactcggtcgccgcatacactattctcagaatgacttggttgagtactcaccagtcacagaaaagcatcttacggatggcatgacagtaaga
gaattatgcagtgctgccataaccatgagtgataacactgcggccaacttacttctgacaacgatcggaggaccgaaggagctaaccgcttttttgcacaacatgggggatcatgtaactcgccttgatcgttgggaaccggagctgaatgaagccataccaaacg
acgagcgtgacaccacgatgcctgtagcaatggcaacaacgttgcgcaaactattaactggcgaactacttactctagcttcccggcaacaattaatagactggatggaggcggataaagttgcaggaccacttctgcgctcggcccttccggctggctggtttattg
ctgataaatctggagccggtgagcgtgggtctcgcggtatcattgcagcactggggccagatggtaagccctcccgtatcgtagttatctacacgacggggagtcaggcaactatggatgaacgaaatagacagatcgctgagataggtgcctcactgattaagcat
tggtaactgtcagaccaagtttactcatatatactttagattgatttaaaacttcatttttaatttaaaaggatctaggtgaagatcctttttgataatctcatgaccaaaatcccttaacgtgagttttcgttccactgagcgtcagaccccgtagaaaagatcaaaggatcttctt
gagatcctttttttctgcgcgtaatctgctgcttgcaaacaaaaaaaccaccgctaccagcggtggtttgtttgccggatcaagagctaccaactctttttccgaaggtaactggcttcagcagagcgcagataccaaatactgtccttctagtgtagccgtagttaggcca
ccacttcaagaactctgtagcaccgcctacatacctcgctctgctaatcctgttaccagtggctgctgccagtggcgataagtcgtgtcttaccgggttggactcaagacgatagttaccggataaggcgcagcggtcgggctgaacggggggttcgtgcacacagcc
cagcttggagcgaacgacctacaccgaactgagatacctacagcgtgagctatgagaaagcgccacgcttcccgaagggagaaaggcggacaggtatccggtaagcggcagggtcggaacaggagagcgcacgagggagcttccagggggaaacgcc
tggtatctttatagtcctgtcgggtttcgccacctctgacttgagcgtcgatttttgtgatgctcgtcaggggggcggagcctatggaaaaacgccagcaacgcggcctttttacggttcctggccttttgctggccttttgctcacatgttctttcctgcgttatcccctgattctgtg
gataaccgtattaccgcctttgagtgagctgataccgctcgccgcagccgaacgaccgagcgcagcgagtcagtgagcgaggaagcggaagagcgcccaatacgcaaaccgcctctccccgcgcgttggccgattcattaatgcagctggcacgacaggtttc
ccgactggaaagcgggcagtgagcgcaacgcaattaatgtgagttagctcactcattaggcaccccaggctttacactttatgcttccggctcgtatgttgtgtggaattgtgagcggataacaatttcacacaggaaacagctatgaccatgattacgccaagctcag
aattaaccctcactaaagggactagtcctgcaggtttaaacgaatttgtggtttggaggtagagtggggtccaagaattaatggtgtccaagaagcctgacttcgctggcacaccaagaagaagggaccagggaagggagatctgagggttcattgggtattaacat
gagattacaggcctgtgtctgtctacgtctgtctctctgatgtgttcgtttgatgactaaatgaggtaatttatgtaaaggcacagcttaggacttattacattccccccccccttttttttctgttttcaaaaccatttatatatatatttttagacctgcaatttataaggagaatagtga
ggagatggccctaagaaaatagcagcttcaccatagtaaaaagacagatggcgctgacaagatgattcagcttgctgccaagactgatgacttaaggttagtcctcagaaccatatggtagaaggagagaactgactcctgcaagttgtccagtggtccctcatgt
gccctgtgccacaggctctccccccacccccaacaaacaagcaaatagatatattcaattaaaaataaaagagagatgaaagagcctgaaaggacactgaaagccctggagaccttaagtgtgctgttgtgcgtagcacagtgatttaattaagatgtgtgtagtct
gtgaatgcacttggctaatgagcatgttgatcgaaagcactggccagtcctgagtaatgtcagattatcaggtgctctaacattctaaattattttctgcactttgaagtagagagtgtagaagcaaaacaagccagtttaagcattaggggttgcatcctttgacaaaagc
tactggaaggtaaggtgtgttacagagcctacagaaatggtttcagattaagtggtcaatgttattttaaagtgcatgcatatatattcagttttaaaatcagttacaggaaagctacaaagggtgctagcattgtacaataaatatatatctcttcagtgtcttgaaatctggta
tgacatgtgactcctggggggctatctgcacaggttctcagtgtttctccagcagcagcagtctctggcttctgtggctatattagaacagtgaactatactatactatgaggctgctcttcgtgctgtggttgactgcagtcatcacagtgtttacaaagatggctgatgagtata
cgtgctcatgtatgctcctgcctgactgtttgctgattgacttcatcaggcatatctgtttctgactaatactggagagagagagtagaaactttcttagtgtaaaggtacccagtgacttgagtcatgacaaactcacagtctgcctagttccatttccctttcccctgtgataccctc
ctggggtgttttactgagtttacatctggtatattctctaagtggcacatggctcccatgcatctatggattcccctgggcaattgattttaaaacagctccttgcatgttgtgcagagatgtggaaattggctactggctgcaggatgaatgcacagcttgaattgaaaatctag
cgaagcagaattctctacttacagctgtgttaattcaaataggacattttaggcctagagagctggtcacatcttctaagggctgtaagtagcctttcttacatgggggtgagttgctttttctttaggaccatctagtcacttttttttttaaatgaattctcaagtataagagaac
atagttttgaaaagtgaaaagtgattttttttaatagtaagaagcttgcttttgttgtagggattttacttcctcgtgtagtgaggtttcttaggtgtatactattgtgcagccattcacttgcactgtcataggcattgggcggtgtgtgtttgaacatgattttgtgcttggatcttaattc
aacttgattggaaatgcccatgtctgcccatgagtttgttttgacctcagtcattcaatggcatgagagagttagtggtctgagggactggagtcatcttagaggagaacacagtgctgtgactgtcacttttgttcgtggcatagtgctttcttcctcttctgttcagaaaagac
ctcttggttattgatagtaactctgggtgatgttttctgatttcataatgaactccagagggcaatgtactggccagtgttaaatgagagtaatatatcagatgttctgtgtacgtgaccatcatatgcctgggacttccttgttttggaaaatcccatttaatgcacagcacatca
ggtagccaaaagaatttcctctcccatcagctaccctgtccctcccaaaccagggagtttatccaccattttctcaccagtcccctcagtctgtttctagatagatgtatctaggctgatgactggaagattctcctgtaggttttgactcatcccatgaatcactcaatcccaat
gcatttacaacagaggcaaaggacagctttcagtggtgccccgtccagatgtcctcgctctgtggagctatctactccctgccaggacccctgagatgcaaagcccactctccctggtagaggctgctgcagcgaaactaaattgtagctgagaaggtgaccacttg
gccatctgaaaagtctgaccggttggcacactgttgtctccaaatgagccggcaggaaacggggtttcctaaaacttggttttaaagcacttagaacaggaaaccatttcccatgcatgttagatctctctaatgaccagctaactagaaacaggctatcttaatactctg
ccccgagccattacaaatggctttctttccagacttcattagaatatttactgcaatttagatagaagacttttgacactgcagtcataaaatttacagggaaccatttttttgtttttttgtttgttttccttttttttttttttcagtctaagaagaacttggaagaagtgaggaaagccct
ggactcccccagccaccaatttcactcattagtggcctttgtgtctcatgacctctagttaagaaaataaacatccctgtggtagtccaataaattgccttcagaaaaaaacagctcgatctctttttgtcaactcctgcctctttcagaattacagcaagcttagtaaggtgc
atatctgagcgactactgacacgtctggctatgctgtttgcttttgttgccacccgtcttagtatccgtgtgtgtatgtgagggaggtaattacatatttagttacactgtctggttttgttggtggtatttggttttgttacatcactcagctttcaggcactatggttctttgatctcatctg
cagagtattatgttaattttctctgtacctataaagaataagggatttctacatataaggctaaattgtctctttatttgaataataatgagttttggactcaagtccagactctccttacctactgtgcctcagatatatttctcaagagtgtttctgtgaagcttgctgccctcacctgt
gttggtaacatgccaaagtgtgctgggagcacagttatgtttagtgttcacacgtgtctagtataagggattgctaatgagacatcaggagtgttgtgagcattactttttcacaccatagcccaggaatcttgtattgagtcaggtaggggcatacacgtgtaattgcagca
gttgggcagctgaaggaggattgcaaggattcggctagcttgatctacaaggtatgaagccaggtctcatggcattgtattgtcatggggcactttgttgatctctcttgaattataaggtctgttttatttagaaaagtacagatcataaaagatgggctgtgctgttttcata
gggaaccaaacctcaggctcatttaataggatgatgaaattaactaacatctctgaatatctcatagttcagtcctctgttaagtgagaagagctttatgagggaagaaataaaaagctactagcttcacatttgaaagcaatatgcttttaattaccatgctcccaatgctt
acattcagaaaacaatcttatagaaataatcattactaattaaaaactttcccctagtttttttgtggtgagtcagggggggagaattgaaaactatctacttttaggttaaaatgaattttctttgcaagtgaggttagatgtcatatttaatgctgctggaggtttcttagggtacagt
actccagaccgactggatgactcaccataccctatgcagtttgagttgtagatctgtgtctatttggcatttaaactcatatgcacttgcctataaaatcattgttcagtatgttgctctggtagcaatttgcagtgtctgtgaaatattagccaatgactctgtgaatctacacagc
tgaaactgtctggctgaaacttggtgcagcatattaactgacagcccattatgtagtttgtatcccaggaatagaagtgacagtgggactgtttgacagcttagtataagtggcatctcaagttgtgatgaactattccagtcactgactatgacaccacctcagacagcct
ctgtgcacaccttcaattaaaagaaaactctagacttaaataaatactgcagtgatgaattttaaacagcagacatattaaactacacatgtgtatgtatcttcttagtggtaaagtaagatcagcaggtaaaaactgaaaaaaagaggggtgcatttataactaa

2. REX-*Shh*-promoter::*lacZ*

PCR4-TOPO backbone
H11 Homology Arms
Enhancer (REX Element)
Shhp::*lacZ*::SV40

gaattcgtttaaacctgcaggactagtccctttagtgagggttaattctgagcttggcgtaatcatggtcatagctgtttcctgtgtgaaattgttatccgctcacaattccacacaacatacgagccggaagcataaagtgtaaagcctggggtgcctaatgagtgagcta
actcacattaattgcgttgcgctcactgcccgctttccagtcgggaaacctgtcgtgccagctgcattaatgaatcggccaacgcgcggggagaggcggtttgcgtattgggcgctcttccgcttcctcgctcactgactcgctgcgctcggtcgttcggctgcggcgagc
ggtatcagctcactcaaaggcggtaatacggttatccacagaatcaggggataacgcaggaaagaacatgtgagcaaaaggccagcaaaagcccaggaaccgtaaaaaggccgcgttgctggcgtttttccataggctccgcccccctgacgagcatcacaa
aaatcgacgctcaagtcagaggtggcgaaacccgacaggactataaagataccaggcgtttccccctggaagctccctcgtgcgctctcctgttccgaccctgccgcttaccggatacctgtccgcctttctcccttcgggaagcgtggcgctttctcatagctcacgct
gtaggtatctcagttcggtgtaggtcgttcgctccaagctgggctgtgtgcacgaaccccccgttcagcccgaccgctgcgccttatccggtaactatcgtcttgagtccaacccggtaagacacgacttatcgccactggcagcagccactggtaacaggattagcag
agcgaggtatgtaggcggtgctacagagttcttgaagtggtggcctaactacggctacactagaaggacagtatttggtatctgcgctctgctgaagccagttaccttcggaaaaagagttggtagctcttgatccggcaaacaaaccaccgctggtagcggtggttttt
ttgtttgcaagcagcagattacgcgcagaaaaaaaggatctcaagaagatcctttgatcttttctacggggtctgacgctcagtggaacgaaaactcacgttaagggattttggtcatgagattatcaaaaaggatcttcacctagatccttttaaattaaaaatgaagtttt
aaatcaatctaaagtatatatgagtaaacttggtctgacagttaccaatgcttaatcagtgaggcacctatctcagcgatctgtctatttcgttcatccatagttgcctgactccccgtcgtgtagataactacgatacgggagggcttaccatctggccccagtgctgcaatg
ataccgcgagacccacgctcaccggctccagatttatcagcaataaaccagccagccggaagggccgagcgcagaagtggtcctgcaactttatccgcctccatccagtctattaattgttgccgggaagctagagtaagtagttcgccagttaatagtttgcgcaa
cgttgttgccattgctacaggcatcgtggtgtcacgctcgtcgtttggtatggcttcattcagctccggttcccaacgatcaaggcgagttacatgatcccccatgttgtgcaaaaaagcggttagctccttcggtcctccgatcgttgtcagaagtaagttggccgcagtgtt
atcactcatggttatggcagcactgcataattctcttactgtcatgccatccgtaagatgcttttctgtgactggtgagtactcaaccaagtcattctgagaatagtgtatgcggcgaccgagttgctcttgcccggcgtcaatacgggataataccgcgccacatagcaga
actttaaaagtgctcatcattggaaaacgttcttcggggcgaaaactctcaaggatcttaccgctgttgagatccagttcgatgtaacccactcgtgcacccaactgatcttcagcatcttttactttcaccagcgtttctgggtgagcaaaaacaggaaggcaaaatgcc
gcaaaaaagggaataagggcgacacggaaatgttgaatactcatactcttcctttttcaatattattgaagcatttatcagggttattgtctcatgagcggatacatatttgaatgtatttagaaaaataaacaaataggggttccgcgcacatttccccgaaaagtgccac
ctgtatgcggtgtgaaataccgcacagatgcgtaaggagaaaataccgcatcaggaaattgtaagcgttaatattcagaagaactcgtcaagaaggcgatagaaggcgatgcgctgcgaatcgggagcggcgataccgtaaagcacgaggaagcggtcag
cccattcgccgccaagctcttcagcaatatcacgggtagccaacgctatgtcctgatagcggtccgccacacccagccggccacagtcgatgaatccagaaaagcggccattttccaccatgatattcggcaagcaggcatcgccatgggtcacgacgagatcct
cgccgtcgggcatgctcgccttgagcctggcgaacagttcggctggcgcgagcccctgatgctcttcgtccagatcatcctgatcgacaagaccggcttccatccgagtacgtgctcgctcgatgcgatgtttcgcttggtggtcgaatgggcaggtagccggatcaa
gcgtatgcagccgccgcattgcatcagccatgatggatactttctcggcaggagcaaggtgagatgacaggagatcctgccccggcacttcgcccaatagcagccagtcccttcccgcttcagtgacaacgtcgagcacagctgcgcaaggaacgcccgtcgtg
gccagccacgatagccgcgctgcctcgtcttgcagttcattcagggcaccggacaggtcggtcttgacaaaaagaaccgggcgcccctgcgctgacagccggaacacggcggcatcagagcagccgattgtctgttgtgcccagtcatagccgaatagcctctc

cacccaagcggccggagaacctgcgtgcaatccatcttgttcaatcatgcgaaacgatcctcatcctgtctcttgatcagagcttgatcccctgcgccatcagatccttggcggcgagaaagccatccagtttactttgcagggcttcccaaccttaccagagggcgcc
ccagctggcaattccggttcgcttgctgtccataaaaccgcccagtctagctatcgccatgtaagcccactgcaagctacctgctttctctttgcgcttgcgttttcccttgtccagatagcccagtagctgacattcatccggggtcagcaccgtttctgcggactggctttcta
cgtgaaaaggatctaggtgaagatcctttttgataatctcatgcctgacatttatattccccagaacatcaggttaatggcgtttttgatgtcattttcgcggtggctgagatcagccacttcttccccgataacggagaccggcacactggccatatcggtggtcatcatgcg
ccagctttcatccccgatatgcaccaccgggtaaagttcacgggagactttatctgacagcagacgtgcactggccagggggatcaccatccgtcgccccggcgtgtcaataatatcactctgtacatccacaaacagacgataacggctctctcttttataggtgtaa
accttaaactgccgtacgtataggctgcgcaactgttgggaagggcgatcggtgcgggcctcttcgctattacgccagctggcgaaagggggatgtgctgcaaggcgattaagttgggtaacgccagggttttcccagtcacgacgttgtaaaacgacggccagtg
aattgtaatacgactcactatagggcgaattgaatttagccctggtacatgtatggtggcaagtgttcaccacatccacctccttgtaagtatccaaatcaacaaaagtagtaaactttgacatttttaggaacatctacaactcatgagtttcttctttctccccatcaggtgtg
cttcctcctgggatagacatggctcctttacaggggctatctggcccactcctgggtcaacccttgtaccctttggtctctgctgctagtcaccctctcctaaaccctcgtcctgggacccctctgcatttggcggtgatgcagcagcagctacagcgctcaggtaaggtttag
aaataggccaggttagctgatcacactggaaagttctgagatttccaggagcaatagttatcactgccccttgtttcttaggtgtcactagaaaaacaggtgaagtgaactgacctgtgcagtctcagggattattactactgcaaggacagtcggagccagccatgct
ctagctctgtttttcacttcttataagcctgagaatttttgctgagatgtgaacatgtcagccttggtgggctttcttacgttgcattttcttacgttgcattttgcactttaggaagcctttggttaaatcctctggtagacttcccatcccccaaagtaacaaaccattcaagcaaagt
aggtcagaaaatattgtcaatactgagaacaggacttgggagtgagtatccaagggtggtcttagtagacttgtggggttctgtgggtgaggtgtagagtagagctactttgctgcactgacactcttctctccatagttctgcatcctccaggctctagttcccaggcagc
agctatcagcgttcagactcctcagaatgtacccagccggtcgggcatgccccacatgcactcccagctggagcatcgtaccagccaaaggagcagctcccctgtgggccttgccaaatggtttggctcagatgtgctacagcagcctctgccctccatgcccacc
aaagtcatcagtgtagatgaactggaatatagacagtgaagagggcaggctggctcacccatacctggacctgtggtgacaccctggtcatgactctcattccctctttgtaatgggcttttacattggagcacactatgtgaagatgtttaggggatccacatacctacc
atgatctacattatgacagaaggctgttaaatcgaatgaacctacatggttcaaatacaagggatacaagattgtcagtcctggaagtctttcttttataaaatatgtgaatgaagtgttggtgtcttctagaggtgacacctaagggttctgaaaaaataaaatgtatagac
ccttatgtacagacctgtgtataaactttgtacatacaaatagggtagctttttttgaacttatacatacagctgtacataaagtaactatcagttaggcttgtgtcaactgtttggattttttcacttgaatatttgggactttttcttttggtttattaaaagttacatatgccacgtgtgt
gaacgatatggctggtactgtgtttatttcttccatgaactaagacagtctaaatgagttcctttcacgttttaattttaccttaggacttctggaatttcttctgcacataaagttctgatagcattagtttaagctggactaaccctgaaagtagcttgtggcaagtatcaaggaat
caatattatactctacaaaatcaaagtttacagagaagtcatatagtaatttttctgaaatttactggcacaatgttaatccagcctgactccaactaattaatggtcacattaatttaagtctttcccttgcctctgctgcattagtttctctcaaaattgttaacttacaacttgaag
tctggtattataaattgaatgtaaagcattctgaaagatactatactgattgcaggtttttcagtcaggttcaagctaatttgaccagtcattggattaattatggatctggggccataaatgctattttaattccactatagagattaaaataagccattctccatttcataatattct
attggactttgactgcaggggcctccaagtcttgacagtagattataatccttcagctgcccactctactggaggaggacaaactggtcacttttcagcaaaacctggctgtggatcagggcagtctggtacttccaagctcattagatgccatcatgctctcactgcctcct
cagcttcaagaggaatctggaaaaagcagtcccactggtcaggaaaggaacactagtgcacttatcctgggtgtctgctgagccaacagtggtagtaaggtaagggcaggatgtgtcaaactgccaatagagaactacttactcttcaggctgaagctgatggaa
cagcggccgcagctattgtactataacaaaccaccccaaaactcagagccttaaaacagcagttatttattctcacgcatctgagggttggctgtggaggctttgcttctcactgcagtctgtgggttagtagaggtgactctgctatatgtgtttctcatcatcctcctggaa
ccagcagactagctggttcttctcaaggtgagagcagagctgcaaaaaggcaggaagaaatgtggcctttgaaggccacggctcagaactggcactgtgtacttttaattatgtgttgctggccaaaagctaaagtaaagtcaagagatatgaaaggaagttctgc
ccaaaatgaggctatggcaaaaatgtggatgaagtgagcagtgaagaattgggccctcaaatgtaatttaccatcaagaataataaaagtactttctcattgcattgccctcacaattagttttgaaagtgtctgtaaagtgcttatgagggctgtgccctctcagataata
gtaggatgcctggaggtgggcccctttactgacctctcagtaaggtcagtaataaggctgcaatcaaaaatttggggctaaggattttcctctttgatttgggcagctcttagagggggtgtcttccgaaggtgaggaaatgtgcagtcttcctggaaccatcaatttcctgctt
ccaagtacagtattggtccttacctcacacaccaaagaaaacaaatggatttgattagcaaatactttcatctacatcctaattgaagtctctgggacttccttgttttggaaaatcccattttaatgcacagcacatcaggtagccaaaagaatttcctctcccatcagctac
cctgtccctcccaaaccagggagtttatccaccagaaagaaaaaatttgcaatgtgtgcaccttccacatgttaagttactgaatcttcaaaaccatcttctaaatgggcacgatcattatccccatttcatctctgagaaaattgagaccagaagtcaccaaaagccac
agaggtagtgagtgacagagctaagattggcatccaagtgacactgaaacacagctctctctgaccgccttcactgcttgtacttatcctcatagcagatcccatgcggccgccaggaacatccaaactgagcagcctttctttctctctttctttctttttaaactggaagc
cttcaggtgaaaataaagccacagcagccagagggggggagaaatgtagtctttctcagggttaacatcagaagacaagcttgtgcggcttggccaatcagatgcgcccctgccagctataatatagtgcgaaaggcagcctgtctcacaagctctccagccttgct
accatttaaaatcaggctctttttgtcttttaattgctgtctcgagacccaactccgatgtgttccgttaccagcgaccggcagcctgccatcgcagccccagtctgggtggggatcggagacaagtcccctgcagcagcggcaggcaaggttatataggaagagaaa
gagccaggcagcgccagagggaacgaacgagccgagcgaggaagggagagccgagcgcaaggaggagcgcacacgcacacacccgcgcgtacccgctcgcgcacagacagcgcggggacagctcacaagtcctcaggttccgcggacgagatg
gctcgcgatgatcccgtcgttttacaacgtcgtgactgggaaaaccctggcgttacccaacttaatcgccttgcagcacatccccctttcgccagctggcgtaatagcgaagaggcccgcaccgatcgcccttcccaacagttgcgcagcctgaatggcgaatggcg
ctttgcctggtttccggcaccagaagcggtgccggaaagctggctggagtgcgatcttcctgaggccgatactgtcgtcgtcccctcaaactggcagatgcacggttacgatgcgcccatctacaccaacgtgacctatcccattacggtcaatccgccgtttgttccca
cggagaatccgacgggttgttactcgctcacatttaatgttgatgaaagctggctacaggaaggccagacgcgaattatttttgatggcgttaactcggcgtttcatctgtggtgcaacgggcgctgggtcggttacggccaggacagtcgtttgccgtctgaatttgacct
gagcgcatttttacgcgccggagaaaaccgcctcgcggtgatggtgctgcgctggagtgacggcagttatctggaagatcaggatatgtggcggatgagcggcattttccgtgacgtctcgttgctgcataaaccgactacacaaatcagcgatttccatgttgccact
cgctttaatgatgatttcagccgcgctgtactggaggctgaagttcagatgtgcggcgagttgcgtgactacctacgggtaacagtttctttatggcagggtgaaacgcaggtcgccagcggcaccgcgcctttcggcggtgaaattatcgatgagcgtggtggttatgc
cgatcgcgtcacactacgtctgaacgtcgaaaacccgaaactgtggagcgccgaaatcccgaatctctatcgtgcggtggttgaactgcacaccgccgacggcacgctgattgaagcagaagcctgcgatgtcggtttccgcgaggtgcggattgaaaatggtct
gctgctgctgaacggcaagccgttgctgattcgaggcgttaaccgtcacgagcatcatcctctgcatggtcaggtcatggatgagcagacgatggtgcaggatatcctgctgatgaagcagaacaactttaacgccgtgcgctgttcgcattatccgaaccatccgct
gtggtacacgctgtgcgaccgctacggcctgtatgtggtggatgaagccaatattgaaacccacggcatggtgccaatgaatcgtctgaccgatgatccgcgctggctaccggcgatgagcgaacgcgtaacgcgaatggtgcagcgcgatcgtaatcacccga
gtgtgatcatctggtcgctggggaatgaatcaggccacggcgctaatcacgacgcgctgtatcgctggatcaaatctgtcgatccttcccgcccggtgcagtatgaaggcggcggagccgacaccacggccaccgatattatttgcccgatgtacgcgcgcgtggat
gaagaccagcccttcccggctgtgccgaaatggtccatcaaaaaatggctttcgctacctggagagacgcgcccgctgatcctttgcgaatacgcccacgcgatgggtaacagtcttggcggtttcgctaaatactggcaggcgtttcgtcagtatccccgtttacagg
gcggcttcgtctgggactgggtggatcagtcgctgattaaatatgatgaaaacggcaacccgtggtcggcttacggcggtgattttggcgatacgccgaacgatcgccagttctgtatgaacggtctggtctttgccgaccgcacgccgcatccagcgctgacggaag
caaaacaccagcagcagtttttccagttccgtttatccgggcaaaccatcgaagtgaccagcgaatacctgttccgtcatagcgataacgagctcctgcactggatggtggcgctggatggtaagccgctggcaagcggtgaagtgcctctggatgtcgctccacaa
ggtaaacagttgattgaactgcctgaactaccgcagccggagagcgccgggcaactctggctcacagtacgcgtagtgcaaccgaacgcgaccgcatggtcagaagccgggcacatcagcgcctggcagcagtggcgtctggcggaaaacctcagtgtgac
gctccccgccgcgtcccacgccatcccgcatctgaccaccagcgaaatggatttttgcatcgagctgggtaataagcgttggcaatttaaccgccagtcaggctttctttcacagatgtggattggcgataaaaaacaactgctgacgccgctgcgcgatcagttcacc
cgtgcaccgctggataacgacattggcgtaagtgaagcgacccgcattgaccctaacgcctgggtcgaacgctggaaggcggcgggccattaccaggccgaagcagcgttgttgcagtgcacggcagatacacttgctgatgcggtgctgattacgaccgctca
cgcgtggcagcatcaggggaaaaccttatttatcagccggaaaacctaccggattgatggtagtggtcaaatggcgattaccgttgatgttgaagtggcgagcgatacaccgcatccggcgcggattggcctgaactgccagctggcgcaggtagcagagcgggt
aaactggctcggattagggccgcaagaaaactatcccgaccgccttactgccgcctgttttgaccgctgggatctgccattgtcagacatgtataccccgtacgtcttcccgagcgaaaacggtctgcgctgcgggacgcgcgaattgaattatggcccacaccagt
ggcgcggcgacttccagttcaacatcagccgctacagtcaacagcaactgatggaaaccagccatcgccatctgctgcacgcggaagaaggcacatggctgaatatcgacggtttccatatggggattggtggcgacgactcctggagcccgtcagtatcggcg
gaattccagctgagcgccggtcgctaccattaccagttggtctggtgtcaaaaataataataaccgggcaggggggatctaagctctagataagtaatgatcataatcagccatatcacatctgtagaggttttacttgctttaaaaaacctcccacacctccccctgaa
cctgaaacataaaatgaatgcaattgttgttgttaacttgtttattgcagcttataatggttacaaataaagcaatagcatcacaaatttcacaaataaagcatttttttcactgcattctagttgtggtttgtccaaactcatcaatgtatcttatcatgtctggatccactagttcta
gagcggcaagcagaaagggaacaaggggatattaaatgtgtatagacacgctagagagatggctcagcagttaagagaactagctggtctttcagaggtcctgagatcaattttagacacccacatggtggctcatgaccatctatctataaatggatctgattttca
tgtctggcagtgtacagaagctaactgaagaaaggtggaagacccacaagagttcaagataagccctatatagtgaagttcaaggcaagctttttctacctgaaacttagtctcaaaaaaaaatgaatacgtaaacagtcttccaggggataagaaccttacagaa
aaagcagaaatgcctggggcactggattaccgatgtaatcaaattcagtccttgaattgaacacaggattgcctagagcaaggccagccagagattcatctcagagggagaaaggtgtctttggagcaattttgtggtaatctagtatgtatcacataagtttagacgc
atttgggactggaaagatgtgaacaaagcaccctatggctcacatctgtcattaactctagttccagtgcacctgacaccgtcttctggcctctgcagtgaccaagcacatgggtagtatgtagacatatacataagcaaaacacacatcattaaaaagtgacatttccc
aaaggaagctgaagaaccagttcttgagaagatagtagaaatcagaaggggaaatagtagacatacagagggactgaccaggttgtgtcacctttataggctaggctaatggatgatcgacactagcgctctttgtgaaggacacacaaatgagacatagtttat
aggactaaacacacttctaagcaatttaatgagacttaagaccctgtctctagcaaatactctggatgatattcagctcaaggctcttgtcagacatgtttccattttcaaggtgagctaactggccaaaactgccaacaacctgtagtgaatagagaagatgagaaaat
ctggattctcaaatgacctaatgaaagccactggagcgccatatggtttctgtgaaaatgccttttcaatcattaacctcttaaatgagtgttagcatcctaactaatgagtggtgcagaatagtgggtctgcttagcttaactaaggccaagaaaacaaaacaggaaatt
cattccatgtcatgagactcatactacgaggttcccttagacctcaggagaaaaagtctttggctgtaagaacacacctcagtggatgtggtagactatgcctttactcttttttttttttttttttttttgggtttttcgagacagggtttctctgtgtagccttggctgtcctggaactc
actttgttgaccaggctggcctcgaactcagagatccgcctgcctctgcctctcaagtgctagaattaaaagtgtgcgccaccactcccagctatatagactatgcctttaatcaggacacttgggaggcaggggggatctcaaattcaattccagacagatgactgaca
aacacacacatacaccattcgtcttttctttttttctttttctttttgaaaacaggtttctctatgcagccctggctttcctagaattcaatctatagacccagctggcctcaaacacagaacctcttgcctctgcctcctcaacactatgactaaaggtgtgaaccaccaccacca
ccacccacctggccaaagaatgaattgagtgaataagtacgagtgcaactctccagcagcctgaggcaagaaagcttaacagttgaacctgagaccctacttcgtttgtgttgtacatatcttctcataatgaactaggcagcctagtctctctaccaaatacaccatg
cacttacacctcctcaaaccaactgcaacagctacacacatttccatggcttgcctgtaataataaattcacaaacttcctctaagtaacttttagtggaaatctaccaggttatgcctatttgtgattgggtgcacagatgactagatgagatgtaatttgggaagtgacatg
caataggaaaaatgaatatataacctataaatagaaaacaccaaggcaagcgtggtgactcacacctttaattagcacttgggaggcagaagtcgcatcatctcttaggtcaaggccaatctagttccagaccagcaaggacaacagaaaccttgccttgatata
aacattttaaaactttaaaaatagagaagcccaaactctgctcattggtaatctacccttcctttctttagacaccttgagaagctaggctacatagctttgggtccttacgtacccccactcactcagcctgcagtcaat

3. HS72-*Shh*-promoter::*lacZ*

pBS backbone
Enhancer (HS72)
Shhp::*lacZ*::SV40

tttctttctctctttctttctttttaaactggaagccttcaggtgaaaataaagccacagcagccagagggggggagaaatgtagtctttctcagggttaacatcagaagacaagcttgtgcggcttggccaatcagatgcgcccctgccagctataatatagtgcgaaagg
cagcctgtctcacaagctctccagccttgctaccatttaaaatcaggctctttttgtcttttaattgctgtctcgagacccaactccgatgtgttccgttaccagcgaccggcagcctgccatcgcagccccagtctgggtggggatcggagacaagtcccctgcagcagc
ggcaggcaaggttatataggaagagaaagagccaggcagcgccagagggaacgaacgagccgagcgaggaagggagagccgagcgcaaggaggagcgcacacgcacacacccgcgcgtacccgctcgcgcacagacagcgcggggacagctc
acaagtcctcaggttccgcggacgagatggctcgcgatgatcccgtcgttttacaacgtcgtgactgggaaaaccctggcgttacccaacttaatcgccttgcagcacatccccctttcgccagctggcgtaatagcgaagaggcccgcaccgatcgcccttcccaa
cagttgcgcagcctgaatggcgaatggcgctttgcctggtttccggcaccagaagcggtgccggaaagctggctggagtgcgatcttcctgaggccgatactgtcgtcgtcccctcaaactggcagatgcacggttacgatgcgcccatctacaccaacgtgaccta
tcccattacggtcaatccgccgtttgttcccacggagaatccgacgggttgttactcgctcacatttaatgttgatgaaagctggctacaggaaggccagacgcgaattatttttgatggcgttaactcggcgtttcatctgtggtgcaacgggcgctgggtcggttacggcc
aggacagtcgtttgccgtctgaatttgacctgagcgcatttttacgcgccggagaaaaccgcctcgcggtgatggtgctgcgctggagtgacggcagttatctggaagatcaggatatgtggcggatgagcggcattttccgtgacgtctcgttgctgcataaaccgact
acacaaatcagcgatttccatgttgccactcgctttaatgatgatttcagccgcgctgtactggaggctgaagttcagatgtgcggcgagttgcgtgactacctacgggtaacagtttctttatggcagggtgaaacgcaggtcgccagcggcaccgcgcctttcggcgg
tgaaattatcgatgagcgtggtggttatgccgatcgcgtcacactacgtctgaacgtcgaaaacccgaaactgtggagcgccgaaatcccgaatctctatcgtgcggtggttgaactgcacaccgccgacggcacgctgattgaagcagaagcctgcgatgtcggtt
tccgcgaggtgcggattgaaaatggtctgctgctgctgaacggcaagccgttgctgattcgaggcgttaaccgtcacgagcatcatcctctgcatggtcaggtcatggatgagcagacgatggtgcaggatatcctgctgatgaagcagaacaactttaacgccgtg
cgctgttcgcattatccgaaccatccgctgtggtacacgctgtgcgaccgctacggcctgtatgtggtggatgaagccaatattgaaacccacggcatggtgccaatgaatcgtctgaccgatgatccgcgctggctaccggcgatgagcgaacgcgtaacgcgaat
ggtgcagcgcgatcgtaatcacccgagtgtgatcatctggtcgctggggaatgaatcaggccacggcgctaatcacgacgcgctgtatcgctggatcaaatctgtcgatccttcccgcccggtgcagtatgaaggcggcggagccgacaccacggccaccgatat
tatttgcccgatgtacgcgcgcgtggatgaagaccagcccttcccggctgtgccgaaatggtccatcaaaaaatggctttcgctacctggagagacgcgcccgctgatcctttgcgaatacgcccacgcgatgggtaacagtcttggcggtttcgctaaatactggca
ggcgtttcgtcagtatccccgtttacagggcggcttcgtctgggactgggtggatcagtcgctgattaaatatgatgaaaacggcaacccgtggtcggcttacggcggtgattttggcgatacgccgaacgatcgccagttctgtatgaacggtctggtctttgccgaccg
cacgccgcatccagcgctgacggaagcaaaacaccagcagcagtttttccagttccgtttatccgggcaaaccatcgaagtgaccagcgaatacctgttccgtcatagcgataacgagctcctgcactggatggtggcgctggatggtaagccgctggcaagcgg
tgaagtgcctctggatgtcgctccacaaggtaaacagttgattgaactgcctgaactaccgcagccggagagcgccgggcaactctggctcacagtacgcgtagtgcaaccgaacgcgaccgcatggtcagaagccgggcacatcagcgcctggcagcagtg
gcgtctggcggaaaacctcagtgtgacgctccccgccgcgtcccacgccatcccgcatctgaccaccagcgaaatggatttttgcatcgagctgggtaataagcgttggcaatttaaccgccagtcaggctttctttcacagatgtggattggcgataaaaaacaact
gctgacgccgctgcgcgatcagttcacccgtgcaccgctggataacgacattggcgtaagtgaagcgacccgcattgaccctaacgcctgggtcgaacgctggaaggcggcgggccattaccaggccgaagcagcgttgttgcagtgcacggcagatacactt
gctgatgcggtgctgattacgaccgctcacgcgtggcagcatcaggggaaaaccttatttatcagccggaaaacctaccggattgatggtagtggtcaaatggcgattaccgttgatgttgaagtggcgagcgatacaccgcatccggcgcggattggcctgaactg
ccagctggcgcaggtagcagagcgggtaaactggctcggattagggccgcaagaaaactatcccgaccgccttactgccgcctgttttgaccgctgggatctgccattgtcagacatgtataccccgtacgtcttcccgagcgaaaacggtctgcgctgcgggacg
cgcgaattgaattatggcccacaccagtggcgcggcgacttccagttcaacatcagccgctacagtcaacagcaactgatggaaaccagccatcgccatctgctgcacgcggaagaaggcacatggctgaatatcgacggtttccatatggggattggtggcga
cgactcctggagcccgtcagtatcggcggaattccagctgagcgccggtcgctaccattaccagttggtctggtgtcaaaaataataataaccgggcaggggggatctaagctctagataagtaatgatcataatcagccatatcacatctgtagaggttttacttgcttt
aaaaaacctcccacacctccccctgaacctgaaacataaaatgaatgcaattgttgttgttaacttgtttattgcagcttataatggttacaaataaagcaatagcatcacaaatttcacaaataaagcatttttttcactgcattctagttgtggtttgtccaaactcatcaatgt
atcttatcatgtctggatccactagttctagagcggccgccaccgcggtgcgtcgacgccaattcgccctatagtgagtcgtattacaattcactggccgtcgttttacaacgtcgtgactgggaaaaccctggcgttacccaacttaatcgccttgcagcacatccccctttt
cgccagctggcgtaatagcgaagaggcccgcaccgatcgcccttcccaacagttgcgcagcctgaatggcgaatggaaattgtaagcgttaatattttgttaaaattcgcgttaaatttttgttaaatcagctcattttttaaccaataggccgaaatcggcaaaatccctt
ataaatcaaaagaatagaccgagatagggttgagtgttgttccagtttggaacaagagtccactattaaagaacgtggactccaacgtcaaagggcgaaaaaccgtctatcagggcgatggcccactacgtgaaccatcaccctaatcaagttttttggggtcgag

gtgccgtaaagcactaaatcggaaccctaaagggagcccccgatttagagcttgacggggaaagccggcgaacgtggcgagaaaggaagggaagaaagcgaaaggagcgggcgctagggcgctggcaagtgtagcggtcacgctgcgcgtaaccacc
acacccgccgcgcttaatgcgccgctacagggcgcgtcaggtggcacttttcggggaaatgtgcgcggaacccctatttgtttatttttctaaatacattcaaatatgtatccgctcatgagacaataaccctgataaatgcttcaataatattgaaaaaggaagagtatg
agtattcaacatttccgtgtcgcccttattcccttttttgcggcattttgccttcctgtttttgctcacccagaaacgctggtgaaagtaaaagatgctgaagatcagttgggtgcacgagtgggttacatcgaactggatctcaacagcggtaagatccttgagagttttcgccc
cgaagaacgttttccaatgatgagcacttttaaagttctgctatgtggcgcggtattatcccgtattgacgccgggcaagagcaactcggtcgccgcatacactattctcagaatgacttggttgagtactcaccagtcacagaaaagcatcttacggatggcatgacag
taagagaattatgcagtgctgccataaccatgagtgataacactgcggccaacttacttctgacaacgatcggaggaccgaaggagctaaccgcttttttgcacaacatgggggatcatgtaactcgccttgatcgttgggaaccggagctgaatgaagccatacca
aacgacgagcgtgacaccacgatgcctgtagcaatggcaacaacgttgcgcaaactattaactggcgaactacttactctagcttcccggcaacaattaatagactggatggaggcggataaagttgcaggaccacttctgcgctcggcccttccggctggctggtt
tattgctgataaatctggagccggtgagcgtgggtctcgcggtatcattgcagcactggggccagatggtaagccctcccgtatcgtagttatctacacgacggggagtcaggcaactatggatgaacgaaatagacagatcgctgagataggtgcctcactgatta
agcattggtaactgtcagaccaagtttactcatatatactttagattgatttaaaacttcatttttaatttaaaaggatctaggtgaagatcctttttgataatctcatgaccaaaatcccttaacgtgagttttcgttccactgagcgtcagaccccgtagaaaagatcaaaggat
cttcttgagatcctttttttctgcgcgtaatctgctgcttgcaaacaaaaaaaccaccgctaccagcggtggtttgtttgccggatcaagagctaccaactctttttccgaaggtaactggcttcagcagagcgcagataccaaatactgttcttctagtgtagccgtagttagg
ccaccacttcaagaactctgtagcaccgcctacatacctcgctctgctaatcctgttaccagtggctgctgccagtggcgataagtcgtgtcttaccgggttggactcaagacgatagttaccggataaggcgcagcggtcgggctgaacggggggttcgtgcacaca
gcccagcttggagcgaacgacctacaccgaactgagatacctacagcgtgagctatgagaaagcgccacgcttcccgaagggagaaaggcggacaggtatccggtaagcggcagggtcggaacaggagagcgcacgagggagcttccagggggaaac
gcctggtatctttatagtcctgtcgggtttcgccacctctgacttgagcgtcgatttttgtgatgctcgtcaggggggcggagcctatggaaaaacgccagcaacgcggcctttttacggttcctggccttttgctggccttttgctcacatgttctttcctgcgttatcccctgattct
gtggataaccgtattaccgcctttgagtgagctgataccgctcgccgcagccgaacgaccgagcgcagcgagtcagtgagcgaggaagcggaagagcgcccaatacgcaaaccgcctctccccgcgcgttggccgattcattaatgcagctggcacgacagg
tttcccgactggaaagcgggcagtgagcgcaacgcaattaatgtgagttagctcactcattaggcaccccaggctttacactttatgcttccggctcgtatgttgtgtggaattgtgagcggataacaatttcacacaggaaacagctatgaccatgattacgccaagct
cgaaattaaccctcactaaagggaacaaaagctggtacctgctgtaattctgaaagaggcaggagttgacaaaaaagagatcgagctgtttttttctgaaggcaatttattggactaccacagggatgtttattttcttaactagaggtcatgagacacaaaggccact
aatgagtgaaattggtggctgggggagtccagggctttcctcacttcttccaagttcttcttagactgaaaaaaaaaaaaaaaaggaaaacaaacaaaaaacaaaaaaaatggttccctgtaaattttatgactgcagtgtcaaaagtcttctatctaaattgcagtaaatatt
ctaatgaagtctggaaagaaagccatttgtaatggctcggggcagagtattaagatagcctgtttctagttagctggtcattagagagatctaacatgcatgggaaatggtttcctgttctaagtgctttaaaaccaagttttaggaaaccccgtttcctgccggctcatttgg
agacaacagtgtgccaaccggtcagacttttcagatggccaagtggtcaccttctcagctacaatttagtttcgctgcagcagcctctaccagggagagtgggctttgcatctcaggggtcctggcagggagtagatagctccacagagcgaggacatctggacggg
gcaccactgaaagctgtcctttgcctctgttgtaaatgcattgggattgagtgattcatgggatgagtcaaaacctacaggagaatcttccagtcatcagcctagatacatctatctagaaacagactgaggggactggtgagaaaatggtggataaactccctggtttg
ggagggacagggtagctgatgggagaggaaattcttttggctacctgatgtgctgtgcattaaaatgggattttccaaaacaaggaagtcccaggtaccgggccccccctcgaggtcgacggtatcgataagcttgatatcgaattcc

4. HS72-*Shh*-promoter::*Shh*

PCR4-TOPO backbone
H11 Homology Arms
Enhancer (HS72)
Shhp::*lacZ*::SV40
Shh

gaattcgtttaaacctgcaggactagtccctttagtgagggttaattctgagcttggcgtaatcatggtcatagctgtttcctgtgtgaaattgttatccgctcacaattccacacaacatacgagccggaagcataaagtgtaaagcctggggtgcctaatgagtgagcta
actcacattaattgcgttgcgctcactgcccgctttccagtcgggaaacctgtcgtgccagctgcattaatgaatcggccaacgcgcggggagaggcggtttgcgtattgggcgctcttccgcttcctcgctcactgactcgctgcgctcggtcgttcggctgcggcgagc
ggtatcagctcactcaaaggcggtaatacggttatccacagaatcaggggataacgcaggaaagaacatgtgagcaaaaggccagcaaaagcccaggaaccgtaaaaaggccgcgttgctggcgtttttccataggctccgcccccctgacgagcatcacaa
aaatcgacgctcaagtcagaggtggcgaaacccgacaggactataaagataccaggcgtttccccctggaagctccctcgtgcgctctcctgttccgaccctgccgcttaccggatacctgtccgcctttctcccttcgggaagcgtggcgctttctcatagctcacgct
gtaggtatctcagttcggtgtaggtcgttcgctccaagctgggctgtgtgcacgaaccccccgttcagcccgaccgctgcgccttatccggtaactatcgtcttgagtccaacccggtaagacacgacttatcgccactggcagcagccactggtaacaggattagcag
agcgaggtatgtaggcggtgctacagagttcttgaagtggtggcctaactacggctacactagaaggacagtatttggtatctgcgctctgctgaagccagttaccttcggaaaaagagttggtagctcttgatccggcaaacaaaccaccgctggtagcggtggtttttt
ttgtttgcaagcagcagattacgcgcagaaaaaaaggatctcaagaagatcctttgatcttttctacggggtctgacgctcagtggaacgaaaactcacgttaagggattttggtcatgagattatcaaaaaggatcttcacctagatccttttaaattaaaaatgaagtttt
aaatcaatctaaagtatatatgagtaaacttggtctgacagttaccaatgcttaatcagtgaggcacctatctcagcgatctgtctatttcgttcatccatagttgcctgactccccgtcgtgtagataactacgatacgggagggcttaccatctggccccagtgctgcaatg
ataccgcgagacccacgctcaccggctccagatttatcagcaataaaccagccagccggaagggccgagcgcagaagtggtcctgcaactttatccgcctccatccagtctattaattgttgccgggaagctagagtaagtagttcgccagttaatagtttgcgcaa
cgttgttgccattgctacaggcatcgtggtgtcacgctcgtcgtttggtatggcttcattcagctccggttcccaacgatcaaggcgagttacatgatcccccatgttgtgcaaaaaagcggttagctccttcggtcctccgatcgttgtcagaagtaagttggccgcagtgtt
atcactcatggttatggcagcactgcataattctcttactgtcatgccatccgtaagatgcttttctgtgactggtgagtactcaaccaagtcattctgagaatagtgtatgcggcgaccgagttgctcttgcccggcgtcaatacgggataataccgcgccacatagcaga
actttaaaagtgctcatcattggaaaacgttcttcggggcgaaaactctcaaggatcttaccgctgttgagatccagttcgatgtaacccactcgtgcacccaactgatcttcagcatcttttactttcaccagcgtttctgggtgagcaaaaacaggaaggcaaaatgcc
gcaaaaaagggaataagggcgacacggaaatgttgaatactcatactcttcctttttcaatattattgaagcatttatcagggttattgtctcatgagcggatacatatttgaatgtatttagaaaaataaacaaataggggttccgcgcacatttccccgaaaagtgccac
ctgtatgcggtgtgaaataccgcacagatgcgtaaggagaaaataccgcatcaggaaattgtaagcgttaataattcagaagaactcgtcaagaaggcgatagaaggcgatgcgctgcgaatcgggagcggcgataccgtaaagcacgaggaagcggtcag
cccattcgccgccaagctcttcagcaatatcacgggtagccaacgctatgtcctgatagcggtccgccacacccagccggccacagtcgatgaatccagaaaagcggccattttccaccatgatattcggcaagcaggcatcgccatgggtcacgacgagatcct
cgccgtcgggcatgctcgccttgagcctggcgaacagttcggctggcgcgagcccctgatgctcttcgtccagatcatcctgatcgacaagaccggcttccatccgagtacgtgctcgctcgatgcgatgtttcgcttggtggtcgaatgggcaggtagccggatcaa
gcgtatgcagccgccgcattgcatcagccatgatggatactttctcggcaggagcaaggtgagatgacaggagatcctgccccggcacttcgcccaatagcagccagtcccttcccgcttcagtgacaacgtcgagcacagctgcgcaaggaacgcccgtcgtg
gccagccacgatagccgcgctgcctcgtcttgcagttcattcagggcaccggacaggtcggtcttgacaaaaagaaccgggcgcccctgcgctgacagccggaacacggcggcatcagagcagccgattgtctgttgtgcccagtcatagccgaatagcctctc
cacccaagcggccggagaacctgcgtgcaatccatcttgttcaatcatgcgaaacgatcctcatcctgtctcttgatcagagcttgatcccctgcgccatcagatccttggcggcgagaaagccatccagtttactttgcagggcttcccaaccttaccagagggcgcc
ccagctggcaattccggttcgcttgctgtccataaaaccgcccagtctagctatcgccatgtaagcccactgcaagctacctgctttctctttgcgcttgcgttttcccttgtccagatagcccagtagctgacattcatccggggtcagcaccgtttctgcggactggctttcta
cgtgaaaaggatctaggtgaagatcctttttgataatctcatgcctgacatttatattccccagaacatcaggttaatggcgtttttgatgtcattttcgcggtggctgagatcagccacttcttccccgataacggagaccggcacactggccatatcggtggtcatcatgcg
ccagctttcatccccgatatgcaccaccgggtaaagttcacgggagactttatctgacagcagacgtgcactggccaggggggatcaccatccgtcgccccggcgtgtcaataatatcactctgtacatccacaaacagacgataacggctctctcttttataggtgtaa
accttaaactgccgtacgtataggctgcgcaactgttgggaagggcgatcggtgcgggcctcttcgctattacgccagctggcgaaagggggatgtgctgcaaggcgattaagttgggtaacgccagggttttcccagtcacgacgttgtaaaacgacggccagtg
aattgtaatacgactcactatagggcgaattgaatttagccctggtacatgtatggtggcaagtgttcaccacatccacctccttgtaagtatccaaatcaacaaaagtagtaaactttgacatttttaggaacatctacaactcatgagtttcttctttctccccatcaggtgtg
cttcctcctgggatagacatggctcctttacaggggctatctggcccactcctgggtcaacccttgtaccctttggtctctgctgctagtcaccctctcctaaaccctcgtcctgggacccctctgcatttggcggtgatgcagcagcagctacagcgctcaggtaaggtttag
aaataggccaggttagctgatcacactggaaagttctgagatttccaggagcaatagttatcactgccccttttgtttcttaggtgtcactagaaaaacaggtgaagtgaactgacctgtgcagtctcagggattattactactgcaaggacagtcggagccagccatgct
ctagctctgtttttcacttcttataagcctgagaatttttgctgagatgtgaacatgtcagccttggtgggctttcttacgttgcattttcttacgttgcattttgcactttaggaagcctttggttaaatcctctggtagacttcccatcccccaaagtaacaaaccattcaagcaaagt
aggtcagaaaatattgtcaatactgagaacaggacttgggagtgagtatccaagggtggtcttagtagacttgtggggttctgtgggtgaggtgtagagtagagctactttgctgcactgacactcttctctccatagttctgcatcctccaggctctagttcccaggcagc
agctatcagcgttcagactcctcagaatgtacccagccggtcgggcatgccccacatgcactcccagctggagcatcgtaccagccaaaggagcagctcccctgtgggccttgccaaatggtttggctcagatgtgctacagcagcctctgccctccatgcccacc
aaagtcatcagtgtagatgaactggaatatagacagtgaagagggcaggctggctcacccatacctggacctgtggtgacaccctggtcatgactctcattccctctttgtaatgggcttttacattggagcacactatgtgaagatgtttaggggatccacatacctacc
atgatctacattatgacagaaggctgttaaatcgaatgaacctacatggttcaaatacaagggatacaagattgtcagtcctggaagtctttcttttataaaatatgtgaatgaagtgttggtgtcttctagaggtgacacctaagggttctgaaaaaataaaatgtatagac
ccttatgtacagacctgtgtataaactttgtacatacaaatagggtagctttttttgaacttatacatacagctgtacataaagtaactatcagttaggcttgtgtcaactgtttggatttttttcacttgaatatttgggacttttttcttttggtttattaaaagttacatatgccacgtgtgt
gaacgatatggctggtactgtgtttatttcttccatgaactaagacagtctaaatgagttcctttcacgttttaattttaccttaggacttctggaatttcttctgcacataaagttctgatagcattagtttaagctggactaaccctgaaagtagcttgtggcaagtatcaaggaat
caatattatactctacaaaatcaaagtttacagagaagtcatatagtaatttttctgaaatttactggcacaatgttaatccagcctgactccaactaattaatggtcacattaatttaagtctttcccttgcctctgctgcattagtttctctcaaaattgttaacttacaacttgaag
tctggtattataaattgaatgtaaagcattctgaaagatactatactgattgcaggtttttcagtcaggttcaagctaatttgaccagtcattggattaattatggatctggggccataaatgctattttaattccactatagagattaaaataagccattctccatttcataatattct
attggactttgactgcaggggcctccaagtcttgacagtagattataatccttcagctgcccactctactggaggaggacaaactggtcacttttcagcaaaacctggctgtggatcagggcagtctggtacttccaagctcattagatgccatcatgctctcactgcctcct
cagcttcaagaggaatctggaaaaagcagtcccactggtcaggaaaggaacactagtgcacttatcctgggtgtctgctgagccaacagtggtagtaaggtaagggcaggatgtgtcaaactgccaatagagaactacttactcttcaggctgaagctgatggaa
cagcggccgcgtaccgggccccccctcgagttttttttttttttttttgtgtgtggcacgctttatttctataaaactattacaaaatattcaaaaatacatttagtaaatttacagcagttacttctcagtttattaatgcaaaaacatccttgttttctctgtacaaactacaggctc
catcctcgtgggctcgctgctaggtgcacttttaaaaaatattattttctaataaattctttgaagttaaaaataacagagttcttccagtctgtcaaaaaaaaaagtgtatatgtgtgcgttaacagtcttggtttccaagaacatgacaaagtggcggttacaaagcaaatac
aaaccaagaaggcagtacagaagacggctattacattggaaaatagttaaattaaaataatatattttcatattttacactttcaaaaaatgaaatgtcattcagttaggtattgatctctcaaaaatctaatttacaattctgtgtataaaaatataatttgtggacccccatg
gagcaggttttagtttacacttgcgggaaagcaggagcatagcaggagaggaatgcggaggtttgccttctgaaacgcaggacaagggacatgtagaaaaatagactttcagcaggttgcttggcctcacaaaataatctttccctcactgtgagtacagttcacac
aataataaattatccaagacacaaactagttaaggctcttgaaggtccgttcatatttttaaaacatctattcatacgaaacaaataaatagccaggagaggagaaaaacaacccatataagacaataacgaaaaatttcaaaaaaatatataaactaaacataac
aacagaactccccggggtttttgcttccttatagtctactttggactgccctacttattattattattattatcattattattattattatgaattattcttataattaaaagtctccttccgtgcgctttcccatcagttccttatttcgcagtcgctccccgccccgcccgccccttgcccggt
cccgtcgggcttcagctggacttgaccgccattcccaagggatgcatggtctcgctgtccaacagccaggtgccaatgtggtagagcagctgcgagtaccagtggatgcccgcagtcggctccgcgcccctcgcttccgttgcagattgcgctgcaggatgctgcc
cccgcccccgccgtccgtgcgggcgggtgccagcgcggccagcagcgcgtgcgccaggcggaaaggcgcgaaggcccggtgtgcccagctgtgctcctcgatgacagcgtagcacgaggcgagcaccccggttgatgagaatggtgccgtgcgccgtgag
cggcgcgtacgcgcccgcctcctcctctcgcagcgtcacgctgtgcaccgcggcgggcagcagccggcggtccccgccgcgttcagccaccacgtacacgcgctgcccggggcgcacgcggctggcaaagagcgcgcttggcccgggcgtgggccccgag
tcgttgtgcggcgccacgaagagcaggtgcgcggcggtgagcagcaggcgctcgcgcggctccagcgtctcgatcacgtagaagaccttcttggcgccttcgtcgcggtccaggaaggtgaggaagtcgctgtacagcagccggccctggtcgtcagccgcca
gcacgcggtctccgggacgtaagtccttcaccagcttggtgccgccctgctccaggtgcacggtggcggatcccgggaaacagccgccggatttggccgccacggagttctctgctttcacagaacagtggatgtgagctttggattcatagtagacccagtcgaaa
cctgcttccacagccaggcgagccagcatgccgtacttgctgcggtcccggtcggacgtggtgatgtccactgctcgaccctcatagtgtagagactcctctgaatgatggccgtcctcatcccagccctcggtcactcgcagcttcactccaggccactggttcatcac
agagatggccaaggcatttaacttgtctttgcacctctgagtcatcagccggtctgctcccgtgttttcctcatccttaaatatgatgtcggggttgtaattgggggtgagttccttaaatcgttcggagtttcttgtgatcttcccttcatatctgccgctggcccctagggtcttctc
ggctacgttgggaataaactgcttgtaggctaaaggggtcagctttttggggtgccgcctctttccaaaccccctgccgggcccacaggccagcccggggcacaccagcagcgaggaagcaaggatcaccagaaaacatctggccagcagcagcagcatctc
gtccgcggaacctgaggacttgtgagctgtccccgcgctgtctgtgcgcgagcgggtacgcgcgggtgtgtgcgtgtgcgctcctccttgcgctcggctctcccttcctcgctcggctcgttcgttccctctggcgctgcctggctctttctcttcctatataaccttgcctgccg
ctgctgcaggggacttgtctccgatccccacccagactggggctgcgatggcaggctgccggtcgctggtaacggaacacatcggagttgggtctcgagacagcaattaaaagacaaaaagagcctgatttaaatggtagcaaggctggagagcttgtgagac
aggctgcctttcgcactatattatagctggcaggggcgcatctgattggccaagccgcacaagcttgtcttctgatgttaaccctgagaaagactacatttctccccccctctggctgctgtggctttattttcacctgaaggcttccagtttaaaaaagaaagaaagagaga
aagaaaggaattcgatatcaagcttatcgataccgtcgacctcgagggggggcccggtacctgggacttccttgttttggaaaatcccattttaatgcacagcacatcaggtagccaaaagaatttcctctcccatcagctaccctgtccctcccaaaccagggagttta
tccaccattttctcaccagtcccctcagtctgtttctagatagatgtatctaggctgatgactggaagattctcctgtaggttttgactcatcccatgaatcactcaatcccaatgcatttacaacagaggcaaaggacagctttcagtggtgccccgtccagatgtcctcgctc
tgtggagctatctactccctgccaggaccccctgagatgcaaagcccactctccctggtagaggctgctgcagcgaaactaaattgtagctgagaaggtgaccacttggccatctgaaaagtctgaccggttggcacactgttgtctccaaatgagccggcaggaaa
cggggtttcctaaaacttggttttaaagcacttagaacaggaaaccatttcccatgcatgttagatctctctaatgaccagctaactagaaacaggctatcttaatactctgccccgagccattacaaatggctttctttccagacttcattagaatatttactgcaatttagata
gaagacttttgacactgcagtcataaaatttacagggaaccatttttttgttttttgtttgttttccttttttttttttcagtctaagaagaacttggaagaagtgaggaaagccctggactcccccagccaccaatttcactcattagtggcctttgtgtctcatgacctctagttaag
aaaataaacatccctgtggtagtccaataaattgccttcagaaaaaaacagctcgatctctttttgtcaactcctgcctctttcagaattacagcagcggccgccaggaacatccaaactgagcagcctttctttctctctttctttctttttaaactggaagccttcaggtga
aaataaagccacagcagccagagggggggagaaatgtagtctttctcagggttaacatcagaagacaagcttgtgcggcttggccaatcagatgcgcccctgccagctataatatagtgcgaaaggcagcctgtctcacaagctctccagccttgctaccatttaaa
atcaggctctttttgtcttttaattgctgtctcgagacccaactccgatgtgttccgttaccagcgaccggcagcctgccatcgcagccccagtctgggtggggatcggagacaagtcccctgcagcagcggcaggcaaggttatataggaagagaaagagccaggc
agcgccagagggaacgaacgagccgagcgaggaagggagagccgagcgcaaggaggagcgcacacgcacacacccgcgcgtacccgctcgcgcacagacagcgcggggacagctcacaagtcctcaggttccgcggacgagatggctcgcgatg
atcccgtcgttttacaacgtcgtgactgggaaaaccctggcgttacccaacttaatcgccttgcagcacatccccctttcgccagctggcgtaatagcgaagaggcccgcaccgatcgcccttcccaacagttgcgcagcctgaatggcgaatggcgctttgcctggtt
tccggcaccagaagcggtgccggaaagctggctggagtgcgatcttcctgaggccgatactgtcgtcgtcccctcaaactggcagatgcacggttacgatgcgcccatctacaccaacgtgacctatcccattacggtcaatccgccgtttgttcccacggagaatc

cgacgggttgttactcgctcacatttaatgttgatgaaagctggctacaggaaggccagacgcgaattatttttgatggcgttaactcggcgtttcatctgtggtgcaacgggcgctgggtcggttacggccaggacagtcgtttgccgtctgaatttgacctgagcgcatttt
tacgcgccggagaaaaccgcctcgcggtgatggtgctgcgctggagtgacggcagttatctggaagatcaggatatgtggcggatgagcggcattttccgtgacgtctcgttgctgcataaaccgactacacaaatcagcgatttccatgttgccactcgctttaatga
tgatttcagccgcgctgtactggaggctgaagttcagatgtgcggcgagttgcgtgactacctacgggtaacagtttctttatggcagggtgaaacgcaggtcgccagcggcaccgcgcctttcggcggtgaaattatcgatgagcgtggtggttatgccgatcgcgtc
acactacgtctgaacgtcgaaaacccgaaactgtggagcgccgaaatcccgaatctctatcgtgcggtggttgaactgcacaccgccgacggcacgctgattgaagcagaagcctgcgatgtcggtttccgcgaggtgcggattgaaaatggtctgctgctgctga
acggcaagccgttgctgattcgaggcgttaaccgtcacgagcatcatcctctgcatggtcaggtcatggatgagcagacgatggtgcaggatatcctgctgatgaagcagaacaactttaacgccgtgcgctgttcgcattatccgaaccatccgctgtggtacacgc
tgtgcgaccgctacggcctgtatgtggtggatgaagccaatattgaaacccacggcatggtgccaatgaatcgtctgaccgatgatccgcgctggctaccggcgatgagcgaacgcgtaacgcgaatggtgcagcgcgatcgtaatcacccgagtgtgatcatctg
gtcgctggggaatgaatcaggccacggcgctaatcacgacgcgctgtatcgctggatcaaatctgtcgatccttcccgcccggtgcagtatgaaggcggcggagccgacaccacggccaccgatattatttgcccgatgtacgcgcgcgtggatgaagaccagc
ccttcccggctgtgccgaaatggtccatcaaaaaatggctttcgctacctggagagacgcgcccgctgatcctttgcgaatacgcccacgcgatgggtaacagtcttggcggtttcgctaaatactggcaggcgtttcgtcagtatccccgtttacagggcggcttcgtct
gggactgggtggatcagtcgctgattaaatatgatgaaaacggcaacccgtggtcggcttacggcggtgattttggcgatacgccgaacgatcgccagttctgtatgaacggtctggtctttgccgaccgcacgccgcatccagcgctgacggaagcaaaacacca
gcagcagtttttccagttccgtttatccgggcaaaccatcgaagtgaccagcgaatacctgttccgtcatagcgataacgagctcctgcactggatggtggcgctggatggtaagccgctggcaagcggtgaagtgcctctggatgtcgctccacaaggtaaacagtt
gattgaactgcctgaactaccgcagccggagagcgccgggcaactctggctcacagtacgcgtagtgcaaccgaacgcgaccgcatggtcagaagccgggcacatcagcgcctggcagcagtggcgtctggcggaaaacctcagtgtgacgctccccgccg
cgtcccacgccatcccgcatctgaccaccagcgaaatggatttttgcatcgagctgggtaataagcgttggcaatttaaccgccagtcaggctttctttcacagatgtggattggcgataaaaaacaactgctgacgccgctgcgcgatcagttcacccgtgcaccgct
ggataacgacattggcgtaagtgaagcgacccgcattgaccctaacgcctgggtcgaacgctggaaggcggcgggccattaccaggccgaagcagcgttgttgcagtgcacggcagatacacttgctgatgcggtgctgattacgaccgctcacgcgtggcag
catcaggggaaaaccttatttatcagccggaaaacctaccggattgatggtagtggtcaaatggcgattaccgttgatgttgaagtggcgagcgatacaccgcatccggcgcggattggcctgaactgccagctggcgcaggtagcagagcgggtaaactggctc
ggattagggccgcaagaaaactatcccgaccgccttactgccgcctgttttgaccgctgggatctgccattgtcagacatgtataccccgtacgtcttcccgagcgaaaacggtctgcgctgcgggacgcgcgaattgaattatggcccacaccagtggcgcggcg
acttccagttcaacatcagccgctacagtcaacagcaactgatggaaaccagccatcgccatctgctgcacgcggaagaaggcacatggctgaatatcgacggtttccatatggggattggtggcgacgactcctggagcccgtcagtatcggcggaattccagct
gagcgccggtcgctaccattaccagttggtctggtgtcaaaaataataataaccgggcaggggggatctaagctctagataagtaatgatcataatcagccatatcacatctgtagaggttttacttgctttaaaaaacctcccacacctccccctgaacctgaaacata
aaatgaatgcaattgttgttgttaacttgtttattgcagcttataatggttacaaataaagcaatagcatcacaaatttcacaaataaagcatttttttcactgcattctagttgtggtttgtccaaactcatcaatgtatcttatcatgtctggatccactagttctagagcggcaag
cagaaagggaacaaggggatattaaatgtgtatagacacgctagagagatggctcagcagttaagagaactagctggtctttcagaggtcctgagatcaattttagacacccacatggtggctcatgaccatctatctataaatggatctgattttcatgtctggcagtg
tacagaagctaactgaagaaaggtggaagacccacaagagttcaagataagccctatatagtgaagttcaaggcaagctttttctacctgaaacttagtctcaaaaaaaaatgaatacgtaaacagtcttccaggggataagaaccttacagaaaaagcagaaa
tgcctggggcactggattaccgatgtaatcaaattcagtccttgaattgaacacaggattgcctagagcaaggccagccagagattcatctcagagggagaaaggtgtctttggagcaattttgtggtaatctagtatgtatcacataagtttagacgcatttgggactgg
aaagatgtgaacaaagcaccctatggctcacatctgtcattaactctagttccagtgcacctgacaccgtcttctggcctctgcagtgaccaagcacatgggtagtatgtagacatatacataagcaaaacacacatcattaaaaagtgacatttcccaaaggaagct
gaagaaccagttcttgagaagatagtagaaatcagaaggggaaatagtagacatacagagggactgaccaggttgtgtcacctttataggctaggctaatggatgatcgacactagcgctctttgtgaaggacacacaaatgagacatagtttataggactaaac
acacttctaagcaatttaatgagacttaagaccctgtctctagcaaatactctggatgatattcagctcaaggctcttgtcagacatgtttccattttcaaggtgagctaactggccaaaactgccaacaacctgtagtgaatagagaagatgagaaaatctggattctca
aatgacctaatgaaagccactggagcgccatatggtttctgtgaaaatgccttttcaatcattaacctcttaaatgagtgttagcatcctaactaatgagtggtgcagaatagtgggtctgcttagcttaactaaggccaagaaacaaaacaggaaattcattccatgtc
atgagactcatactacgaggttcccttagacctcaggagaaaaagtctttggctgtaagaacacacctcagtggatgtggtagactatgcctttactcttttttttttttttttttttttttgggtttttcgagacagggtttctctgtgtagccttggctgtcctggaactcactttgttgac
caggctggcctcgaactcagagatccgcctgcctctgcctctcaagtgctagaattaaaagtgtgcgccaccactcccagctatatagactatgcctttaatcaggacacttgggaggcaggggatctcaaattcaattccagacagatgactgacaaacacacac
atacaccattcgtcttttctttttttctttttcttttgaaaacaggtttctctatgcagccctggctttcctagaattcaatctatagacccagctggcctcaaacacagaacctcttgcctctgcctcctcaacactatgactaaaggtgtgaaccaccaccaccaccacccacct
ggccaaagaatgaattgagtgaataagtacgagtgcaactctccagcagcctgaggcaagaaagcttaacagttgaacctgagaccctacttcgtttgtgttgtacatatcttctcataatgaactaggcagcctagtctctctaccaaatacaccatgcacttacacct
cctcaaaccaactgcaacagctacacacatttccatggcttgcctgtaataataaattcacaaacttcctctaagtaacttttagtggaaatctaccaggttatgcctatttgtgattgggtgcacagatgactagatgagatgtaatttgggaagtgacatgcaataggaa
aaatgaatatataacctataaatagaaaacaccaaggcaagcgtggtgactcacacctttaattagcacttgggaggcagaagtcgcatcatctcttaggtcaaggccaatctagttccagaccagcaaggacaacagaaaccttgccttgatataaacattttaaa
actttaaaaatagagaagcccaaactctgctcattggtaatctacccttcctttctttagacaccttgagaagctaggctacatagctttgggtccttacgtaccccccactcactcagcctgcagtc

4. ZRS_mCH/ZRSΔHD_eGFP-SuperIns

PCR4-TOPO backbone
H11 Homology Arms
Fluorescent Protein (mCherry/eGFP)
Enhancer (ZRS/ZRSΔHD)
SuperInsulator

gaattcgtttaaacctgcaggactagtccctttagtgagggttaattctgagcttggcgtaatcatggtcatagctgtttcctgtgtgaaattgttatccgctcacaattccacacaacatacgagccggaagcataaagtgtaaagcctggggtgcctaatgagtgagcta
actcacattaattgcgttgcgctcactgcccgctttccagtcgggaaacctgtcgtgccagctgcattaatgaatcggccaacgcgcggggagaggcggtttgcgtattgggcgctcttccgcttcctcgctcactgactcgctgcgctcggtcgttcggctgcggcgagc
ggtatcagctcactcaaaggcggtaatacggttatccacagaatcaggggataacgcaggaaagaacatgtgagcaaaaggccagcaaaaggccaggaaccgtaaaaaggccgcgttgctggcgtttttccataggctccgcccccctgacgagcatcacaa
aaatcgacgctcaagtcagaggtggcgaaacccgacaggactataaagataccaggcgtttccccctggaagctccctcgtgcgctctcctgttccgaccctgccgcttaccggatacctgtccgcctttctcccttcgggaagcgtggcgctttctcatagctcacgct
gtaggtatctcagttcggtgtaggtcgttcgctccaagctgggctgtgtgcacgaaccccccgttcagcccgaccgctgcgccttatccggtaactatcgtcttgagtccaacccggtaagacacgacttatcgccactggcagcagccactggtaacaggattagcag
agcgaggtatgtaggcggtgctacagagttcttgaagtggtggcctaactacggctacactagaaggacagtatttggtatctgcgctctgctgaagccagttaccttcggaaaaagagttggtagctcttgatccggcaaacaaaccaccgctggtagcggtggttttt
ttgtttgcaagcagcagattacgcgcagaaaaaaaggatctcaagaagatcctttgatcttttctacggggtctgacgctcagtggaacgaaaactcacgttaagggattttggtcatgagattatcaaaaaggatcttcacctagatccttttaaattaaaaatgaagtttt
aaatcaatctaaagtatatatgagtaaacttggtctgacagttaccaatgcttaatcagtgaggcacctatctcagcgatctgtctatttcgttcatccatagttgcctgactccccgtcgtgtagataactacgatacgggagggcttaccatctggccccagtgctgcaatg
ataccgcgagacccacgctcaccggctccagatttatcagcaataaaccagccagccggaagggccgagcgcagaagtggtcctgcaactttatccgcctccatccagtctattaattgttgccgggaagctagagtaagtagttcgccagttaatagtttgcgcaa
cgttgttgccattgctacaggcatcgtggtgtcacgctcgtcgtttggtatggcttcattcagctccggttcccaacgatcaaggcgagttacatgatcccccatgttgtgcaaaaaagcggttagctccttcggtcctccgatcgttgtcagaagtaagttggccgcagtgtt
atcactcatggttatggcagcactgcataattctcttactgtcatgccatccgtaagatgcttttctgtgactggtgagtactcaaccaagtcattctgagaatagtgtatgcggcgaccgagttgctcttgcccggcgtcaatacgggataataccgcgccacatagcaga
actttaaaagtgctcatcattggaaaacgttcttcggggcgaaaactctcaaggatcttaccgctgttgagatccagttcgatgtaacccactcgtgcacccaactgatcttcagcatcttttactttcaccagcgtttctgggtgagcaaaaacaggaaggcaaaatgcc
gcaaaaaagggaataagggcgacacggaaatgttgaatactcatactcttcctttttcaatattattgaagcatttatcagggttattgtctcatgagcggatacatatttgaatgtatttagaaaaataaacaaataggggttccgcgcacatttccccgaaaagtgccac
ctgtatgcggtgtgaaataccgcacagatgcgtaaggagaaaataccgcatcaggaaattgtaagcgttaataattcagaagaactcgtcaagaaggcgatagaaggcgatgcgctgcgaatcgggagcggcgataccgtaaagcacgaggaagcggtcag
cccattcgccgccaagctcttcagcaatatcacgggtagccaacgctatgtcctgatagcggtccgccacacccagccggccacagtcgatgaatccagaaaagcggccattttccaccatgatattcggcaagcaggcatcgccatgggtcacgacgagatcct
cgccgtcgggcatgctcgccttgagcctggcgaacagttcggctggcgcgagcccctgatgctcttcgtccagatcatcctgatcgacaagaccggcttccatccgagtacgtgctcgctcgatgcgatgtttcgcttggtggtcgaatgggcaggtagccggatcaa
gcgtatgcagccgccgcattgcatcagccatgatggatactttctcggcaggagcaaggtgagatgacaggagatcctgccccggcacttcgcccaatagcagccagtcccttcccgcttcagtgacaacgtcgagcacagctgcgcaaggaacgcccgtcgtg
gccagccacgatagccgcgctgcctcgtcttgcagttcattcagggcaccggacaggtcggtcttgacaaaaagaaccgggcgcccctgcgctgacagccggaacacggcggcatcagagcagccgattgtctgttgtgcccagtcatagccgaatagcctctc
cacccaagcggccggagaacctgcgtgcaatccatcttgttcaatcatgcgaaacgatcctcatcctgtctcttgatcagagcttgatcccctgcgccatcagatccttggcggcgagaaagccatccagtttactttgcagggcttcccaaccttaccagagggcgcc
ccagctggcaattccggttcgcttgctgtccataaaaccgcccagtctagctatcgccatgtaagcccactgcaagctacctgctttctctttgcgcttgcgttttcccttgtccagatagcccagtagctgacattcatccggggtcagcaccgtttctgcggactggctttcta
cgtgaaaaggatctaggtgaagatcctttttgataatctcatgcctgacatttatattccccagaacatcaggttaatggcgtttttgatgtcattttcgcggtggctgagatcagccacttcttccccgataacggagaccggcacactggccatatcggtggtcatcatgcg
ccagctttcatccccgatatgcaccaccgggtaaagttcacgggagactttatctgacagcagacgtgcactggccagggggatcaccatccgtcgccccggcgtgtcaataatatcactctgtacatccacaaacagacgataacggctctctcttttataggtgtaa
accttaaactgccgtacgtataggctgcgcaactgttgggaagggcgatcggtgcgggcctcttcgctattacgccagctggcgaaagggggatgtgctgcaaggcgattaagttgggtaacgccagggttttcccagtcacgacgttgtaaaacgacggccagtg
aattgtaatacgactcactatagggcgaattgaatttagccctggtacatgtatggtggcaagtgttcaccacatccacctccttgtaagtatccaaatcaacaaaagtagtaaactttgacatttttaggaacatctacaactcatgagtttcttctttctccccatcaggtgtg
cttcctcctgggatagacatggctcctttacaggggctatctggcccactcctgggtcaacccttgtaccctttggtctctgctgctagtcaccctctcctaaaccctcgtcctgggacccctctgcatttggcggtgatgcagcagcagctacagcgctcaggtaaggtttag
aaataggccaggttagctgatcacactggaaagttctgagatttccaggagcaatagttatcactgccccttttgtttcttaggtgtcactagaaaaacaggtgaagtgaactgacctgtgcagtctcagggattattactactgcaaggacagtcggagccagccatgct
ctagctctgtttttcacttcttataagcctgagaatttttgctgagatgtgaacatgtcagccttggtgggctttcttacgttgcattttcttacgttgcattttgcactttaggaagcctttggttaaatcctctggtagacttcccatcccccaaagtaacaaaccattcaagcaaagt
aggtcagaaaatattgtcaatactgagaacaggacttgggagtgagtatccaagggtggtcttagtagacttgtggggttctgtgggtgaggtgtagagtagagctactttgctgcactgacactcttctctccatagttctgcatcctccaggctctagttcccaggcagc
agctatcagcgttcagactcctcagaatgtacccagccggtcgggcatgccccacatgcactcccagctggagcatcgtaccagccaaaggagcagctcccctgtgggccttgccaaatggtttggctcagatgtgctacagcagcctctgccctccatgcccacc
aaagtcatcagtgtagatgaactggaatatagacagtgaagagggcaggctggctcacccatacctggacctgtggtgacaccctggtcatgactctcattccctctttgtaatgggcttttacattggagcacactatgtgaagatgtttaggggatccacatacctacc
atgatctacattatgacagaaggctgttaaatcgaatgaacctacatggttcaaatacaagggatacaagattgtcagtcctggaagtctttcttttataaaatatgtgaatgaagtgttggtgtcttctagaggtgacacctaagggttctgaaaaaataaaatgtatagac
ccttatgtacagacctgtgtataaactttgtacatacaaatagggtagcttttttgaacttatacatacagctgtacataaagtaactatcagttaggcttgtgtcaactgtttggatttttttcacttgaatatttgggactttttcttttggtttattaaaagttacatatgccacgtgtgt
gaacgatatggctggtactgtgtttatttcttccatgaactaagacagtctaaatgagttcctttcacgttttaattttaccttaggacttctggaatttcttctgcacataaagttctgatagcattagtttaagctggactaaccctgaaagtagcttgtggcaagtatcaaggaat
caatattatactctacaaaatcaaagtttacagagaagtcatatagtaatttttctgaaatttactggcacaatgttaatccagcctgactccaactaattaatggtcacattaatttaagtctttcccttgcctctgctgcattagtttctctcaaaattgttaacttacaacttgaag
tctggtattataaattgaatgtaaagcattctgaaagatactatactgattgcaggtttttcagtcaggttcaagctaatttgaccagtcattggattaattatggatctggggccataaatgctattttaattccactatagagattaaaataagccattctccatttcataatattct
attggactttgactgcaggggcctccaagtcttgacagtagattataatccttcagctgcccactctactggaggaggacaaactggtcacttttcagcaaaacctggctgtggatcagggcagtctggtacttccaagctcattagatgccatcatgctctcactgcctcct
cagcttcaagaggaatctggaaaaagcagtcccactggtcaggaaaggaacactagtgcacttatcctgggtgtctgctgagccaacagtggtagtaaggtaagggcaggatgtgtcaaactgccaatagagaactacttactcttcaggctgaagctgatggaa
cagccgctctagaactagtggatccagacatgataagatacattgatgagtttggacaaaccacaactagaatgcagtgaaaaaaatgctttatttgtgaaatttgtgatgctattgctttatttgtaaccattataagctgcaataaacaagttaacaacaacaattgcatt
cattttatgtttcaggttcagggggaggtgtgggaggttttttaaagcaagtaaaacctctacagatgtgatatggctgattatgatcattacttatctagagcttagatccccccctgccctagttattattacttgtacagctcgtccatgccgccggtggagtggcggccctcg
gcgcgttcgtactgttccacgatggtgtagtcctcgttgtgggaggtgatgtccaacttgatgttgacgttgtaggcgccgggcagctgcacgggcttcttggccttgtaggtggtcttgacctcagcgtcgtagtggccgccgtccttcagcttcagcctctgcttgatctcgc
ccttcagggcgccgtcctcggggtacatccgctcggaggaggcctcccagcccatggtcttcttctgcattacggggccgtcggaggggaagttggtgccgcgcagcttcaccttgtagatgaactcgccgtcctgcagggaggagtcctgggtcacggtcaccacg
ccgccgtcctcgaagttcatcacgcgctcccacttgaagccctcggggaaggacagcttcaagtagtcggggatgtcggcggggtgcttcacgtaggccttggagccgtacatgaactgaggggacaggatgtcccaggcgaagggcagggggccacccttgg
tcaccttcagcttggcggtctgggtgccctcgtaggggcggccctcgccctcgccctcgatctcgaactcgtggccgttcacggagccctccatgtgcaccttgaagcgcatgaactccttgatgatggccatgttatcctcctcgcccttgctcaccatcgccgcgctctg
cttctggaaggctgcgctccgcggcgtggatgctccggggaaagttgcgctccgcggcagggatgctcctgggaaggttgcgctccgcggcagggatgctctggggaaggctggtcctggccgaggatcgggaacgcgccgctcgctctgcttctcttgtcttcgctt
gtctctggatggaaccagatttggttctgagtagctgtcagcgtctggtgacctgctcgccgccctgcgcctttaaggagtcttcaccggccccgcccactctccgctgggccaatcagcgagccggaggaggccttggggccaggaatcttccagcagtttcgcgtct
ggtggagcttccccgcctcccttgagtaatcggagttgtgggttccgcccttgtccagaactctccagaggtttctggggttcactggagagtacggattcctgagggggagggtgtggggaagtgctggtgctactagtgacactgttgctatggcgacgcattactaag
gcctgtgtggaatggacaagaaagatcacctctagctcggtgttgtgtacagtttgttgtgatttgtggggtttcgccaactcgcacagttctgaatatggggttaaaggctaaaacttaagggctaaaacttctccccgccaagtttaggagacccagggagatgcctg
ggggcgtgtccggtgacgtgatcctctccaatcgcgttacaatggcagtgctgcctctgacctcatggactaatttaggaactagaggctctgtcccagcacaggctcaaagttgccgggaggggcggggtgggggtgggggggacccggctgctcagtttggat
gttcctggcggccgcaagggtttaactccctcacacacacgcatggtactaagaaggtggcaacagacttatttctatttactcatttcatttacctcactcctttagaagcttaagtttgagtttaagtcacaatctttgtaaatctgaacaatcatcaaatgaaatgtcacatg
ttcttttccttgtgatcagttcccctgagttcctgcatgcattccagatgtgcaaagttcaagaaaccgcacacccacccgtcacagaagaacagcgctaccgtggcttgaaaacggccgatcctgtccttactatttctggaaagaaaccagtgctccctagtgggga
gagcagagagttctgattaatacatgactacatctttttcttgcaggtgttgggagaatcaaattaacacatagcaacagttagtgagatatgacttcattttctgtaataaatgccaagatcaaaacatgcccaagtcaaatttccttgcaaggttcccagcaggagccttc
ctcttgcctgtgatttcctttcacccagcagaaccagactgagtttgcgcatttgccactaacactaagcagcacttcctgaatcgctcatttccaacaatttatggatcatcagtggcaaaaaaaacaagcaaaaataatgaaagaatccaatgaacgctcatggagtc
ccaggctggacttcctactctcttctctttaagatggaggcctgagacaaattagccactgaggggaaaagtcatctggtcataaaatacagtacaaggtcacttttatgtaagtttgccaaaagcgacataaaccaggacaatctcaaactgtgacacaggatagaa

acatattaaaacgatcttagttcctcctctattgtgctgtcatgttgcttggctttatggacagcctgagcccagcaaagtgcttcgcctccacctggtcagtgcaactaattaagagaggaagtgacctcagattttgtgcattttacttttattatgaaagtactttgtttgaagtt
acaggagctaacctcttaatcctatagatcatgtgtgaggttctggacactctaggatggctggatggtttggataattggtcaggatgttgctgtcattatgttaagttttatgccaggacttcaaatcaaagataggcattaaagtaaagggcaatataaatctgctaccag
aaaaagtcctgggctttatgaattaatctgtcgtcatatttacattacacttacaaaacaatcatatggtgagatattttccccactttcattaagttaattttgtctctataaggttttaattcaaagcggccgcagctagtcaggtagtaaaaggtctcaactaggcagtggcag
agcaggattcaaattcagggctgttgtgatgcctccgcagactctgagcgccacctggtggtaatttgtctgtgcctcttctgacgtggaagaacagcaactaacacactaacacggcatttactatgggccagccattgttctaattaatcgccttgcagcacatcccccct
agctagtcaggtagtaaaaggtctcaactaggcagtggcagagcaggattcaaattcagggctgttgtgatgcctccgcagactctgagcgccacctggtggtaatttgtctgtgcctcttctgacgtggaagaacagcaactaacacactaacacggcatttactatg
ggccagccattgttctaaactgccctccgcctggtcagcggcccggactgatgccctggagtgcttgtccgcccactccccaatgtcccccaagatctgttcctccccattcccaggcagagatgagcacagggcacctgcattcctacgtgtgaatcatccgtgtgaa
tcactaaacagtggagagttgcacttggtcataaatcacacgtgactgcagccacacgcaggtgagtcgcacacgcattccagcacatgtcaaatggtcaggaacagctccctgtcaccccatacaccgatccccccacgcatggccctctttgtccccagcctttg
gcacgcccgacctcattctctgctctggagtagggcaggtcaggaaagcgaaatacagcgccggcaaacagggtctgaatgccccgcgtggcattctttccgctccaggaccgccctgggcctgcaggatcctgggcgggagcccaggtgtccgggatctgggc
cactagggactggggaggaacctctcagagaagcccatagcccgcagcggccccgcgcggccggttccggcgccgcactgttccagcctctactatggtacagtccctgcgtcgcagcctcggcgggggctctaagaacgggaggcagaaaaagctcaatc
agcagcaggcgagcttcacccgctgcttccaaatctgtgccaaaatattctatgctgcacagataaaatcctctgtcggttctacaagcctggcttttcctatagagaaccctcttataagcaaaaagtaaagctctcgtgaagagcccggatagctcagtcggtagag
catcagacttttaatctgagggtccagggttcaagtccctgttcgggcggcatgtctttgcttttgggtaccgcacttcgcataaaatggtaacaaaaatagttactagtttttaaagctaggtgatacagaaacttgatttccacaaaatacagcaaacgacggtggaggg
taataaacatactttaaaacagtacaaacgcgagccgtattatcgaatttacattttctggtagccacattacaaagaataaaaagcaagcgaaattaatttcacggatctattttacttaccctaatataccaaaatcttattttaacatataatcaacattctaaaaatatta
atgaaattttaagtctttttgtttgtacaaagccttccttcaaaagaagatgtgtatttagaagctggcaatacatctgaattcatatatttcaagtgctcgataacggtgtctaccatatccaacagcagactttgttttgttttcgttttaagtgacgggtgtggaagacacgcaa
aatagaatgtccagtaatgtacacaatcttgaaagacaccacgagggaggttccatggtgtaatggttagcactctggactctgaatccagcgatccgagttcaaatctcggtggaaccttgaagcttttcttttaatatcagcatgttgaatattgttaattgattttcacaga
ggggatactcctatgacccaagataatacgacaaaaaaaaaaagtaaactaggacactaccaattatttgtgcttttcctaccggataattgagtcctataacacagtgttaagagaacgtacaataaattatttaaatatgttggaagttgagctaacatttgaaaacat
gttaaaatagagatgttatgaagaaacgaaataatagagatgttatgaagaaacgaaatattgcttcagcagttaccatcaggtcttagcttctgggatggatgcaggcctatgatctttcctggaaggtcctggaagggggcgtccgcgggagctcacggggagag
ccccccccccaaagcccccagggctgtaattgcgtccctctcccgctagggggcagcagcgagccgcccggggctccgctccggcccgacgctccccccgcatccccgagccggcagcgtgcggggacagcccgggcacggggaaggtggcacgggatcg
ctttcctctgaacgcttctcgctgctctttgagcctgcagacacctgggggatacggggaaaaagctttaggctgaaagagagatttagaatgacagaatcatagaatggcctgggttggaaaggcccacaatgctcatccagttccaacccccctgctatgtgcagg
gtttcgccagctggcgtaatagcgaagatttcctggaaggtcctggaagggggcgtccgcgggagctcacggggagagccccccccccaaagcccccagggctgtaattgcgtccctctcccgctagggggcagcagcgagccgcccggggctccgctccggc
ccgacgctccccccgcatccccgagccggcagcgtgcggggacagcccgggcacggggaaggtggcacgggatcgctttcctctgaacgcttctcgctgctctttgagcctgcagacacctgggggatacggggaaaaagctttaggctgaaagagagattt
agaatgacagaatcatagaatggcctgggttggaaaggcccacaatgctcatccagttccaacccccctgctatgtgcaggtaccggttttgaattaaaaccttatagagacaaaattaacttaatgaaagtggggaaaatatctcaccatatgattgttttgtaagtgt
aatgtaaatatgacgacagattaattcataaagcccaggactttttctggtagcagatttatattgccctttactttaatgcctatctttgatttgaagtcctggcataaaacttaacataatgacagcaacatcctcgacgtctgtagaaaccatccagccatcctagagtgtc
cagaacctcacacatgatctataggattaagaggttagctcctgtaacttcaaacaaagtactttcataataaaagtaaaatgcacaaaatctgaggtcacttcctctcgacggacactgcactgaccaggtggaggcgaagcactttgctgggctcaggctgtccat
aaagccaagcaacatgacagcacaatagaggaggaactaagatcgttttaatatgtttctatcctgtgtcacagtttgagattgtcctggtttatgtcgcttttggcaaacttacataaaagtgaccttgtactgtattttatgaccagatgacttttcccctcagtagggactgc
tcttcaggcctccatcttaaagagaagagagtaggaagtccagcctgggactccatgagcgttcattggattctttcattatttttgcttgtttttttgccactgatgatccataaattgttggaaatgagcgattcaggaagtgctgcttagtgttagtggcaaatgcgcaaact
cagtctggttctgctgggtgaaaggaaatcacaggcaagaggaaggctcctgctgggaaccttgcaaggaaatttgacttgggcatgttttgatcttggcatttattacagaaaatgaagtcatatctcactaactgttgctatgtgttaatttgattctcccaacacctgcaa
gaaaaagatgtagtcatgtattaatcagaactctctgctctcccccactagggagcactggtttctttccagaaatagtaaggacaggatcggccgttttcaagccacggtagcgctgttcttctgtgacgggtgggtgtgcggtttcttgaactttgcacatctggaatgcat
gcaggaactcaggggaactgatcacaaggaaaagaacatgtgacatttcatttgatgattgttcagatttacaaagattgtgacttaaactcaaacttaagcttctaaaggagtgaggtaaatgaaatgagtaaatagaaataagtctgttgccaccttcttagtaccat
gcgtgtgtgtgagggagttaaacccttaccggtcaggaacatccaaactgagcagccggggtcccccccacccccccaccccgcccctcccggcaactttgagcctgtgctgggacagagcctctagttcctaaattagtccatgaggtcagaggcagcactgccat
tgtaacgcgattggagaggatcacgtcaccggacacgcccccaggcatctccctgggtctcctaaacttggcggggagaagttttagcccttaagttttagcctttaacccccatattcagaactgtgcgagttggcgaaaccccacaaatcacaacaaactgtacac
aacaccgagctagaggtgatctttcttgtccattccacacaggccttagtaatgcgtcgccatagcaacagtgtcactagtagcaccagcacttccccacaccctcccctcaggaatccgtactctccagtgaaccccagaaacctctggagagttctggacaagg
gcggaacccacaactccgattactcaagggaggcggggaagctccaccagacgcgaaactgctggaagattcctggccccaaggcctcctccggctcgctgattggcccagcggagagtgggcggggccggtgaagactccttaaaggcgcagggcggcg
agcaggtcaccagacgctgacagctactcagaaccaaatctggttccatccagagacaagcgaagacaagagaagcagagcgagcggcgcgttcccgatcctcggccaggaccagccttccccagagcatccctgccgcggagcgcaaccttcccagga
gcatccctgccgcggagcgcaactttccccggagcatccacgccgcggagcgcagccttccagaagcagagcgcggcggccaccatggtgagcaagggcgaggagctgttcaccggggtggtgcccatcctggtcgagctggacggcgacgtaaacggcc
acaagttcagcgtgtccggcgagggcgagggcgatgccacctacggcaagctgaccctgaagttcatctgcaccaccggaagctgcccgtgccctggcccaccctcgtgaccaccctgacctacggcgtgcagtgcttcagccgctaccccgaccacatgaag
cagcacgacttcttcaagtccgccatgcccgaaggctacgtccaggagcgcaccatcttcttcaaggacgacggcaactacaagacccgcgccgaggtgaagttcgagggcgacaccctggtgaaccgcatcgagctgaagggcatcgacttcaaggagga
cggcaacatcctggggcacaagctggagtacaactacaacagccacaacgtctatatcatggccgacaagcagaagaacggcatcaaggtgaacttcaagatccgccacaacatcgaggacggcagcgtgcagctcgccgaccactaccagcagaacac
ccccatcggcgacggccccgtgctgctgcccgacaaccactacctgagcacccagtccgccctgagcaaagaccccaacgagaagcgcgatcacatggtcctgctggagttcgtgaccgccgccgggatcactctcggcatggacgagctgtacaagtaata
actagggcagggggggatctaagctctagataagtaatgatcataatcagccatatcacatctgtagaggttttacttgctttaaaaaacctcccacacctccccctgaacctgaaacataaaatgaatgcaattgttgttgttaacttgtttattgcagcttataatggttaca
aataaagcaatagcatcacaaatttcacaaataaagcatttttttcactgcattctagttgtggtttgtccaaactcatcaatgtatcttatcatgtctggatccactagttctagagcggcaagcagaaagggaacaaggggatattaaatgtgtatagacacgctagaga
gatggctcagcagttaagagaactagctggtctttcagaggtcctgagatcaattttagacacccacatggtggctcatgaccatctatctataaatggatctgattttcatgtctggcagtgtacagaagctaactgaagaaaggtggaagacccacaagagttcaag
ataagccctatatagtgaagttcaaggcaagctttttctacctgaaacttagtctcaaaaaaaaatgaatacgtaaacagtcttccaggggataagaaccttacagaaaaagcagaaatgcctggggcactggattaccgatgtaatcaaattcagtccttgaattga
acacaggattgcctagagcaaggccagccagagattcatctcagagggagaaaggtgtctttggagcaattttgtggtaatctagtatgtatcacataagtttagacgcatttgggactggaaagatgtgaacaaagcaccctatggctcacatctgtcattaactctag
ttccagtgcacctgacaccgtcttctggcctctgcagtgaccaagcacatgggtagtatgtagacatatacataagcaaaacacacatcattaaaaagtgacatttcccaaaggaagctgaagaaccagttcttgagaagatagtagaaatcagaaggggaaata
gtagacatacagagggactgaccaggttgtgtcacctttataggctaggctaatggatgatcgacactagcgctctttgtgaaggacacacaaatgagacatagtttataggactaaacacacttctaagcaatttaatgagacttaagaccctgtctctagcaaatact
ctggatgatattcagctcaaggctcttgtcagacatgtttccattttcaaggtgagctaactggccaaaactgccaacaacctgtagtgaatagagaagatgagaaaatctggattctcaaatgacctaatgaaagccactggagcgccatatggtttctgtgaaaatgc
cttttcaatcattaacctcttaaatgagtgttagcatcctaactaatgagtggtgcagaatagtgggtctgcttagcttaactaaggccaagaaaacaaaacaggaaattcattccatgtcatgagactcatactacgaggttcccttagacctcaggagaaaaagtctttg
gctgtaagaacacacctcagtggatgtggtagactatgcctttactcttttttttttttttttttttttgggtttttcgagacagggtttctctgtgtagccttggctgtcctggaactcactttgttgaccaggctggcctcgaactcagagatccgcctgcctctgcctctcaagtgcta
gaattaaaagtgtgcgccaccactcccagctatatagactatgcctttaatcaggacacttgggaggcagggggatctcaaattcaattccagacagatgactgacaaacacacacatacaccattcgtcttttcttttttttctttttttcttttgaaaacaggtttctctatgcag
ccctggctttcctagaattcaatctatagacccagctggcctcaaacacagaacctcttgcctctgcctcctcaacactatgactaaaggtgtgaaccaccaccaccaccacccacctggccaaagaatgaattgagtgaataagtacgagtgcaactctccagcag
cctgaggcaagaaagcttaacagttgaacctgagaccctacttcgtttgtgttgtacatatcttctcataatgaactaggcagcctagtctctctaccaaatacaccatgcacttacacctcctcaaaccaactgcaacagctacacacatttccatggcttgcctgtaata
ataaattcacaaacttcctctaagtaacttttagtggaaatctaccaggttatgcctatttgtgattgggtgcacagatgactagatgagatgtaatttgggaagtgacatgcaataggaaaaatgaatatataacctataaatagaaaacaccaaggcaagcgtggtga
ctcacacctttaattagcacttgggaggcagaagtcgcatcatctcttaggtcaaggccaatctagttccagaccagcaaggacaacagaaaccttgccttgatataaacattttaaaactttaaaaatagagaagcccaaactctgctcattggtaatctacccttccttt
ctttagacaccttgagaagctaggctacatagctttgggtccttacgtacccccactcactcagcctgcagtc

Shhprox-MHA-PCR4-HS72 (Targeting Vector)

PCR4-TOPO backbone

Shh Homology Arms

Enhancer

tttaaaacttcatttttaatttaaaaggatctaggtgaagatcctttttgataatctcatgaccaaaatcccttaacgtgagttttcgttccactgagcgtcagaccccgtagaaaagatcaaaggatcttcttgagatcctttttttctgcgcgtaatctgctgcttgcaaacaaaa
aaaccaccgctaccagcggtggtttgtttgccggatcaagagctaccaactctttttccgaaggtaactggcttcagcagagcgcagataccaaatactgtccttctagtgtagccgtagttaggccaccacttcaagaactctgtagcaccgcctacatacctcgctct
gctaatcctgttaccagtggctgctgccagtggcgataagtcgtgtcttaccgggttggactcaagacgatagttaccggataaggcgcagcggtcgggctgaacggggggttcgtgcacacagcccagcttggagcgaacgacctacaccgaactgagatacct
acagcgtgagctatgagaaagcgccacgcttcccgaagggagaaaggcggacaggtatccggtaagcggcagggtcggaacaggagagcgcacgagggagcttccagggggaaacgcctggtatctttatagtcctgtcgggtttcgccacctctgacttga
gcgtcgatttttgtgatgctcgtcaggggggcggagcctatggaaaaacgccagcaacgcggcctttttacggttcctgggcttttgctggccttttgctcacatgttctttcctgcgttatcccctgattctgtggataaccgtattaccgcctttgagtgagctgataccgctcg
ccgcagccgaacgaccgagcgcagcgagtcagtgagcgaggaagcggaagagcgcccaatacgcaaaccgcctctccccgcgcgttggccgattcattaatgcagctggcacgacaggtttcccgactggaaagcgggcagtgagcgcaacgcaattaat
gtgagttagctcactcattaggcaccccaggctttacactttatgcttccggctcgtatgttgtgtggaattgtgagcggataacaatttcacacaggaaacagctatgaccatgattacgccaagctcagaattaaccctcactaaaggactagtcctgcaggtttaaa
cgaattAGGAATCTACATTACAACTGGTACAGATTCCAGACACAATGGGAGCCACAGTGGCTCTGGAGCCATTGTAATACCTCAACAGCCTGGAACCTGCGAAGGATGAATGGGCAC
AGAACAAGGCTCCGTGACACATCATGGCACTCACATGCCAGATAAATCTGGGTGCCCCATACAGAGTTGGCCCTATACCTCATACGTCAGCATCCAGTCCTCCTTGCTCTTCTTTC
TGGACAGACACCTAGATGATGTAATACCCTATTGCTCAGCCTGTCTGACAGGAGGCTCTGGTCAGAGAGAATCAAAGCAGGTACTGAGGCTCCCTAAATAAGAAGGTAAGAAGTG
TATAATGGTCTCTGACCTCCATTTCCCAGTGTGAGGGAGTTGAAGGCCATCCAAAGCCAGGTACCCCTTTGCACACCCTGATGCATCTGGGCGTCCAGACTTGTGCTCACTCTGG
ACAGAGTGGCTTTTCACAGCTTCCTGCCTTCTCCCCTGGCTTTTGCTCTACACCCCAAACCTCCCGATTTAATGTACCTCCTCACCCGCACACGGACCCCTCACTATACATGCTCTA
GAAAAATATATGAAACTCATTAAGCTTACTACCAGTCCTTTGCTCTGTCTAATAAAGCCATATGTGTTGTTCCTTGCCAGGAGTGGCCATAGAATAATATGTCCAGGGACCCAGGAG
CATCCAGAAGGGACTGAAGAACATCCCCAGGAGCAGAAACCTGCAGCCAAGTGGCTCCCCTCCCCTGTCCCAAGTCTTGGGGATTCCCATCAAGGGAGTCAGCAAAGCAGAAAC
TCTGATGTGATTGTTTGTGAGCACAGCCCTGTAGCTGTTGGCTGACATGATTTTTTTTTTCAAAGCTCCCCAGGTGATTCTAATGCTTAGGCTTAATGGATTACCTCTCCCGGGGGC
ATCTGCGCTTCAGCAGCTCCAAGACTGGAGGAAAGGTGGTGGCATGTTGGAGTGCCAAAGTACCTATGGTAAAGAAATGCCCAGACCTGGGTCAGAAGAACTAGCTATGGCATC
CAGACCCATCCCTTGGTCCTCTGGGCCATAAGGCCACCTGCCTACACTGAGTTTCTCTTTTCAGCCACAAATGTAGCTGGTGGCCCTGTAGAGGACGGAAATCATTTGGAGACTA
CAAAGATTCACAGAACTGAGGCCTGAATTAAATATCCATGGCTTACACCTTACACAGGGACTACTGAAGGTTGGAGAGGGCGTGGACAAAGTATCTCACAGCCATGTGATTCTCTG
GCTGTTAACACAGAAGCAGCTTTCCCAGGCTTAGGGCTTCTCCTTGGCTGTGAGGTTTTCCTCTCCCCTAGATATCACCCCAGAGAACAAAATGGAGCAAGTGAAACTCTATTTGC
CAAGATTGAAAAAAAAATCAGGAAGAAAATGAAACTTAACTGGGTGATTGGATAGAATGAGATGGTGCAGAGCTGTTCTAAGGAAAAGAAATCAACTGGGAGAAAAGAAAAGAGGC
CAGCAGGCAGGGCTGGGCTGGGATCCCTAGTTCAAACTGGGGAAAGGAAATTAAGGGTTGGAAAGGAAAGACAAGTGTCAGAGAGCAAGGAGGAGCCAAAGAGCCTAGTCCTG
CTTCCAGGCCCTTCCAACTGGAGACTGCAGAATTTCCCCAACCGGGCAGGCCAGGGCCGAAGGCAGAGTGAGGACGCCATCTAGCGGCAGGGCCAGAAGGGGGAAGGGGCCA
TGCCCAGGACTATGCCTCCAGGGGTGTGGGGCAGAGTCAAAGGTTGGCCAGTGCTCCTCAGAGTGAAGGAAAGAACGCTGGACGGAAAGAAACAAGAAGAGTGAGAGCCTGCA
AATGCCCAGCAGGAGGAATAGCTGAACCTACACAAATGAGAAGTAGCCTCTGGGAGTTAAATGGCTGGCCCCACCAGGCAGAGATTGTGCTCTGGAACCTCTTACAGGCACAGG
GAGGGGCCCAAATGGTCTAGAGGGGAAAGCAAAGAGAGGGAGCCATCTGGTGTCTGGAGGTGGAAGGCAGCGAGTGTCTGGTGTCTGGGAAGTGGGGAGAGGGTGGGATTCC
CCGGGAAGGAGGCAGAGAATAAAGGCCACATTTCTTCCTGGGACTTCCTTGTTTTGGAAAATCCCATTTTAATGCACAGCACATCAGGTAGCCAAAAGAATTTCCTCTCCCATCAG
CTACCCTGTCCCTCCCAAACCAGGGAGTTTATCCACCATTTTCTCACCAGTCCCCTCAGTCTGTTTCTAGATAGATGTATCTAGGCTGATGACTGGAAGATTCTCCTGTAGGTTTTG
ACTCATCCCATGAATCACTCAATCCCAATGCATTTACAACAGAGGCAAAGGACAGCTTTCAGTGGTGCCCCGTCCAGATGTCCTCGCTCTGTGGAGCTATCTACTCCCTGCCAGGA
CCCCTGAGATGCAAAGCCCACTCTCCCTGGTAGAGGCTGCTGCAGCGAAACTAAATTGTAGCTGAGAAGGTGACCACTTGGCCATCTGAAAAGTCTGACCGGTTGGCACACTGTT
GTCTCCAAATGAGCCGGCAGGAAACGGGGTTTCCTAAAACTTGGTTTTAAAGCACTTAGAACAGGAAACCATTTCCCATGCATGTTAGATCTCTCTAATGACCAGCTAACTAGAAAC
AGGCTATCTTAATACTCTGCCCCGAGCCATTACAAATGGCTTTCTTTCCAGACTTCATTAGAATATTTACTGCAATTTAGATAGAAGACTTTTGACACTGCAGTCATAAAATTTACAG
GGAACCATTTTTTTGTTTTTTGTTTGTTTTCCTTTTTTTTTTTTTTTCAGTCTAAGAAGAACTTGGAAGAAGTGAGGAAAGCCCTGGACTCCCCCAGCCACCAATTTCACTCATTAGTGG
CCTTTGTGTCTCATGACCTCTAGTTAAGAAAATAAACATCCCTGTGGTAGTCCAATAAATTGCCTTCAGAAAAAAACAGCTCGATCTCTTTTTTGTCAACTCCTGCCTCTTTCAGAATT
ACAGCAAGCCAGCCTCATGATCCCGTTCTCAAGACAACAAATATGAACTGTGATTGCTTTTATCCCAGCATTTCTGCAACCTGGAACAGGAAGCAAGCTGTCCCTGGGTACGAGGA
AAAAAACAACAGGGTTTATAGTAAATCTTTACTTTCCTTCTAAAGATCCTTCCAGACTCCAGGAAGGCCGGCCCTTATCAGGCTCTCTTGGAATTAGCATCCATTGTCCCTAGAGAT

GGAGTGGGGGTGAGCAATGGGCCAGAGCAGAACCAAGTCACCTCCTTCTTCACAGCCAAAAGGGAGGTGACCATGTCTGGGCACTCCAAGGCCAGCTGTGCCCACATAGAACA
CTGGCAGCTTAGGGGTTTGTTTCCAAATCACATCTGCAGGTATTCCAGGTCTCCAGGTTACAGAAATGCTTGGATAAAAGCAATCACAGTTCATATTTGTTGTCTTGAGAACAGGAT
CATGAGCCTGACCACAGGGAGAAATGTGGCCTTTATTTTCTGCCTCCTTCCCCAGGAAATCCCACCCACTCCCCACTGCCAATCCAAAGCTGACTAGAGCTGTCTACAGTTGCATG
ATCCATTCACCAGTCTATATTCAAAGACATCAAGAAGAGAGTGCTGGTCAGCATACACAGAGAACAAGGAAATACTGAAGCAAAATGAGGCTCCAGGATGCTCAAGTCTTGTCTAG
CCCACCACCGGCTAGTAATATGAAAGCCACATGCCAGGCAGGCCCACTCTTCTGGATCAAGAACAGTGGGTCCCCTTTGTATCCATTTTTTCTAGTTGATGGGGGATGTACACAGA
GGATACAGGCTGGAGAGTCTGGCTCTTATTTGGTCTTCGTGAAGCTGGGACCAACTCCCACTAAGAATCTGTACTTGGTCCTCATAAATCAAAGGATAGAAAACTCCAGAGAGGGC
AGGTGTGCGGGCTTTAGTCAACCCTTCCAAGCCTGGGATGGTGAGGCTCTGCCATACTGCCATTGAGACTCACAGTAGACAGTAGCAAGCACTTTCCTCGCCTCACACTAACTCA
AAAGCAACCAAGTTTCTAAGTCCTCTGAAAACCCCACTGCCAGCTGAGCTTCAGGTTTGCTGGGCTCTCCCTGCACTGGGACAGGATCAAGAGCCAGAGGCTGGTCTCATGGGAT
AACACTGGAGGACTCGGTCCCTAAGCAGGTATTCCATTCCACATAAATAGTTCGCCCATCCTAGAGGAATCTTGCCTGTGAGTAGGATGGGAGAAAAGCCAGAGCAGATTTCTATG
ATCTATAAAAACAAAGCGTTTCACATTCTATTTTTCTGGTTCAACTAAGACTTTTCTGGAAATTTTTCTTTTTCCTGAAAGAGTGTTGGACACCCCAAGAGTAGTGACCTCTAGACAGC
ATATCCTCTTCTAAGATGCCCCTTATGGCCAGAACACTGTCCTCTAGATCCCTAACACCTTCAGTCTGCAGGCCCTACACCTAGCCAGCTTCTTTCCTTGAAAATTAGCTTGCAAAG
CTCCTGTGAAATACAACTGTGTTAAGGAGCCTGAATTCCACATCAACTTACCCCAGCCCTGCACTTTGCTAGGATAGATTGGAAGGGGTTCTTCACGCATGCAGCCTTGCCCACTT
TCAGTTGCCCCAGGCAGATCACCTCTACCCACCAAGCACACACCAGCTGCCCTCCATGCTGGGGTGCAGCACATAGACCCACTGAACCTATGTACCCCACCACATTCTGCAGACA
CTCTTTACCTGCCCCTCAGCTGGGATGCATCCATCCACCTGCAGAGGCTTGAATGTGGTGCAGTCAGTGAGATACAATAAAGGACAGTATTTTAGGGCACTTAAAACAGCAACTCT
TTAGGTTGTGGCCACCTGTGATTATCCTCTATCTAAAGATGCTAAATATTCAGGAGATGCTCAGGTGAGCTGTTTTCACAACCCCGTGACGTCTGTCCTGTACACTCACATCTGGG
GATGGGGTGTAAATAAGGCAAACAGGAGGGAGGCAGCGTTGCCCTCAAACAATTGAGAGGCTGTTTTATGGTCAAAGCTTGTGGGGGGGGGGGGAGCATCTGGACTGTCCTTTA
AAAACTTATGCTGGCTTTAACCAAAGCATCTTCCATTTAGAGAAAGTAAGGGGGAAGCAGCCCTGAGCACAAAGCAGGCAGTGCGGGGTATTTTTCCAATAAACAGGCCTCCTCCC
TCCCACCCACCTTTACCTTAGTAAAGACTTTCTTTCTTTCTTTCTTTCTTTCTTTCTTTCTTTCTTTCTTTCTTTCTTTCTTTCTTTCTCTCTTTCTTTCTTTTTAAACTGGAAGCCTTCA
GGTGAAAATAAAGCCACAGCAGCCAGAGGGGGGAGAAATGTAGTCTTTCTCAGGGTTAACATCAGAAGACAAGCTTGTGCGGCTTGGCCAATCAGATGCGCCCCTGCCAGCTAT
AATAgctaaattcaattcgccctatagtgagtcgtattacaattcactggccgtcgttttacaacgtcgtgactgggaaaaccctggcgttacccaacttaatcgccttgcagcacatccccctttcgccagctggcgtaatagcgaagaggcccgcaccgatcgccct
tcccaacagttgcgcagcctatacgtacggcagtttaaggtttacacctataaaagagagagccgttatcgtctgtttgtggatgtacagagtgatattattgacacgccggggcgacggatggtgatccccctggccagtgcacgtctgctgtcagataaagtctcccg
tgaactttacccggtggtgcatatcggggatgaaagctggcgcatgatgaccaccgatatggccagtgtgccggtctccgttatcggggaagaagtggctgatctcagccaccgcgaaaatgacatcaaaaacgccattaacctgatgttctggggaatataaatgt
caggcatgagattatcaaaaaggatcttcacctagatccttttcacgtagaaagccagtccgcagaaacggtgctgaccccggatgaatgtcagctactgggctatctggacaagggaaaacgcaagcgcaaagagaaagcaggtagcttgcagtgggcttaca
tggcgatagctagactgggcggttttatggacagcaagcgaaccggaattgccagctggggcgccctctggtaaggttgggaagccctgcaaagtaaactggatggctttctcgccgccaaggatctgatggcgcaggggatcaagctctgatcaagagacagg
atgaggatcgtttcgcatgattgaacaagatggattgcacgcaggttctccggccgcttgggtggagaggctattcggctatgactgggcacaacagacaatcggctgctctgatgccgccgtgttccggctgtcagcgcaggggcgcccggttctttttgtcaagacc
gacctgtccggtgccctgaatgaactgcaagacgaggcagcgcggctatcgtggctggccacgacgggcgttccttgcgcagctgtgctcgacgttgtcactgaagcgggaagggactggctgctattgggcgaagtgccggggcaggatctcctgtcatctcacc
ttgctcctgccgagaaagtatccatcatggctgatgcaatgcggcggctgcatacgcttgatccggctacctgcccattcgaccaccaagcgaaacatcgcatcgagcgagcacgtactcggatggaagccggtcttgtcgatcaggatgatctggacgaagagca
tcaggggctcgcgccagccgaactgttcgccaggctcaaggcgagcatgcccgacggcgaggatctcgtcgtgacccatggcgatgcctgcttgccgaatatcatggtggaaaatggccgcttttctggattcatcgactgtggccggctgggtgtggcggaccgct
atcaggacatagcgttggctacccgtgatattgctgaagagcttggcggcgaatgggctgaccgcttcctcgtgctttacggtatcgccgctcccgattcgcagcgcatcgccttctatcgccttcttgacgagttcttctgaattattaacgcttacaatttcctgatgcggtat
tttctccttacgcatctgtgcggtatttcacaccgcatacaggtggcacttttcggggaaatgtgcgcggaacccctatttgtttatttttctaaatacattcaaatatgtatccgctcatgagacaataaccctgataaatgcttcaataatattgaaaaaggaagagtatgagt
attcaacatttccgtgtcgcccttattcccttttttgcggcattttgccttcctgtttttgctcacccagaaacgctggtgaaagtaaaagatgctgaagatcagttgggtgcacgagtgggttacatcgaactggatctcaacagcggtaagatccttgagagttttcgccccga
agaacgttttccaatgatgagcacttttaaagttctgctatgtggcgcggtattatcccgtattgacgccgggcaagagcaactcggtcgccgcatacactattctcagaatgacttggttgagtactcaccagtcacagaaaagcatcttacggatggcatgacagtaa
gagaattatgcagtgctgccataaccatgagtgataacactgcggccaacttacttctgacaacgatcggaggaccgaaggagctaaccgcttttttgcacaacatgggggatcatgtaactcgccttgatcgttgggaaccggagctgaatgaagccataccaaa
cgacgagcgtgacaccacgatgcctgtagcaatggcaacaacgttgcgcaaactattaactggcgaactacttactctagcttcccggcaacaattaatagactggatggaggcggataaagttgcaggaccacttctgcgctcggcccttccggctggctggtttat
tgctgataaatctggagccggtgagcgtgggtctcgcggtatcattgcagcactggggccagatggtaagccctcccgtatcgtagttatctacacgacggggagtcaggcaactatggatgaacgaaatagacagatcgctgagataggtgcctcactgattaagc
attggtaactgtcagaccaagtttactcatatatactttagattga

Supplementary Table Legends

Table S1. Barcode Metadata for Single-Cell Multiomics. All meta-data outlined for each barcode, including the number of UMI, genes, ATAC fragments, nucleosome signal, transcription start site (TSS) enrichment, MACS2-calculated peaks, and cell type annotation.

Table S2. Long-Range Motif Enrichment Results. TF motifs enriched in functionally validated and putative long-range enhancers. FDR, false-discovery rate; Fold change (long range / short range motif instance).

Table S3. Enhancer Sequences used for generating knock-in mice.

Table S4. Primer sequences.

Table S5. Source data for qPCR

Table S6. Source data measurements from fluorescent images.
