# Supplementary material for: Range extender mediates long-distance enhancer activity
Source: Nature. 2025 Jul 2;643(8072):830–8. doi: 10.1038/s41586-025-09221-6 (PMC12267059; doi:10.1038/s41586-025-09221-6)
Supplement: Supplementary file 2 — Reporting Summary [file 41586_2025_9221_MOESM2_ESM.pdf]

nature portfolio

Corresponding author(s): Evgeny Kvon

Last updated by author(s): Jan 31, 2025

Reporting Summary

Nature Portfolio wishes to improve the reproducibility of the work that we publish. This form provides structure for consistency and transparency in reporting. For further information on Nature Portfolio policies, see our Editorial Policies and the Editorial Policy Checklist.

Statistics

For all statistical analyses, confirm that the following items are present in the figure legend, table legend, main text, or Methods section.

n/a	Confirmed	
☐	☒	The exact sample size (*n*) for each experimental group/condition, given as a discrete number and unit of measurement
☐	☒	A statement on whether measurements were taken from distinct samples or whether the same sample was measured repeatedly
☐	☒	The statistical test(s) used AND whether they are one- or two-sided<br>*Only common tests should be described solely by name; describe more complex techniques in the Methods section.*
☒	☐	A description of all covariates tested
☐	☒	A description of any assumptions or corrections, such as tests of normality and adjustment for multiple comparisons
☐	☒	A full description of the statistical parameters including central tendency (e.g. means) or other basic estimates (e.g. regression coefficient) AND variation (e.g. standard deviation) or associated estimates of uncertainty (e.g. confidence intervals)
☐	☒	For null hypothesis testing, the test statistic (e.g. *F*, *t*, *r*) with confidence intervals, effect sizes, degrees of freedom and *P* value noted<br>*Give P values as exact values whenever suitable.*
☒	☐	For Bayesian analysis, information on the choice of priors and Markov chain Monte Carlo settings
☒	☐	For hierarchical and complex designs, identification of the appropriate level for tests and full reporting of outcomes
☒	☐	Estimates of effect sizes (e.g. Cohen's *d*, Pearson's *r*), indicating how they were calculated

Our web collection on statistics for biologists contains articles on many of the points above.

Software and code

Policy information about availability of computer code

Data collection	Sequencing data were collected on the Illumina NovaSeq 6000. Imaging data were captured through the Zeiss BioLite software (Zen Blue 3.2). qPCR data was collected using a C1000 Touch Thermal Cycler.
Data analysis	Custom algorithms or software was not developed for this research. Fluorescent images were analyzed with Fiji/ImageJ (2.14.0/1.54f). Data were processed using trim_galore (v0.6.4), bedtools (v2.31.1), CellRanger ARC (v2.0.2), and HOMER (v4.11). Data analysis was primarily performed in R (v. >4.1.2) using a variety of published packages: Seurat (v4.4.0), Signac (v1.11.0), GenomicScores (v3.19), rstatix (v0.7.2). qPCR data was processed using CFX Maestro Software (v2.3 Bio-Rad) to extract cycle threshold values.

For manuscripts utilizing custom algorithms or software that are central to the research but not yet described in published literature, software must be made available to editors and reviewers. We strongly encourage code deposition in a community repository (e.g. GitHub). See the Nature Portfolio guidelines for submitting code & software for further information.

Data

Policy information about availability of data

All manuscripts must include a data availability statement. This statement should provide the following information, where applicable:

- Accession codes, unique identifiers, or web links for publicly available datasets
- A description of any restrictions on data availability
- For clinical datasets or third party data, please ensure that the statement adheres to our policy

All sequencing data generated in this study is available at the NCBI Gene Expression Omnibus (GEO) under accession number GSE243635. Capture Hi-C data from a previous study is available at GEO: GSE217078. The mm10 genome assembly and PhyloP are available at UCSC genome browser (https://hgdownload.cse.ucsc.edu/goldenpath/mm10/).

Research involving human participants, their data, or biological material

Policy information about studies with human participants or human data. See also policy information about sex, gender (identity/presentation), and sexual orientation and race, ethnicity and racism.

Reporting on sex and gender	n/a
Reporting on race, ethnicity, or other socially relevant groupings	n/a
Population characteristics	n/a
Recruitment	n/a
Ethics oversight	n/a

Note that full information on the approval of the study protocol must also be provided in the manuscript.

Field-specific reporting

Please select the one below that is the best fit for your research. If you are not sure, read the appropriate sections before making your selection.

☒ Life sciences ☐ Behavioural & social sciences ☐ Ecological, evolutionary & environmental sciences

For a reference copy of the document with all sections, see nature.com/documents/nr-reporting-summary-flat.pdf

Life sciences study design

All studies must disclose on these points even when the disclosure is negative.

Sample size	No prior analyses were used to determine the sample size before the experiment. Embryos litters were collected until at least 2 embryos of the desired genotype were acquired to show that a phenotype was reproducible. For experiments where statisical analysis was conducted (fluroescent imaging and qPCR) embryos were collected until a minimum of three replicates for each group was reached to allow for comparisons.
Data exclusions	Any embryos that were not at the correct developmental stage were excluded from data collection
Replication	ATAC-seq, ISH, skeletal staining, and florescent imaging experiments were completed with at least two biological replicates. For qPCR at least three biological replicates per each comparison group were collected. For the scATAC-seq/scRNA-seq analysis we used one biological replicate.
Randomization	For ATAC-seq, ISH, and skeletal staining experiments wild-type and knockin littermates were identified by numbers with genotype unknown to the investigator during data collection and sample processing. Group assignment was defined based on the mouse genotype so randomized allocation was not necessary.
Blinding	For ISH experiments in knockin embryos, investigators were blinded to animals' genotypes during tissue collection and in situ hybridization. For skeletal staining investigators were blinded to animals' genotypes during embryo/neonate collection and processing. For fluorescent imaging, investigators were blinded to embryos genotypes during imaging. For sequencing experiments, motif enrichment analysis, and qPCR blinding was not performed because all metrics were derived from absolute quantitative measurements without human subjectivity.

Reporting for specific materials, systems and methods

We require information from authors about some types of materials, experimental systems and methods used in many studies. Here, indicate whether each material, system or method listed is relevant to your study. If you are not sure if a list item applies to your research, read the appropriate section before selecting a response.

Materials & experimental systems

n/a	Involved in the study
☐	☒ Antibodies
☒	☐ Eukaryotic cell lines
☒	☐ Palaeontology and archaeology
☐	☒ Animals and other organisms
☒	☐ Clinical data
☒	☐ Dual use research of concern
☒	☐ Plants

Methods

n/a	Involved in the study
☒	☐ ChIP-seq
☒	☐ Flow cytometry
☒	☐ MRI-based neuroimaging

Antibodies

Antibodies used	Sheep Anti-Digoxigenin Fab fragments Antibody, AP Conjugated (www.sigmaaldrich.com/US/en/product/roche/11093274910)<br>Supplier: Roche Cat# 11093274910; Lot No. 54732420
Validation	Online databases: www.antibodyregistry.org/AB_514497<br><br>This is not a monoclonal, but a polyclonal Ab made in sheep, so there is no clone name.<br>After immunization with digoxigenin, sheep IgG was purified by ion-exchange chromatography, and the specific IgG was isolated by immunosorption. The Fab fragments obtained by papain digestion were purified by gel filtration, conjugated to the specific label, and stabilized in buffer. The polyclonal antibody from sheep is specific to digoxigenin and digoxin and shows no cross-reactivity with other steroids, such as human estrogens and androgens. Heat inactivation: yes<br><br>• Cross reactivity to digitoxin and digitoxigenin: <1 %<br>• No cross reactivity with other human estrogen or androgen steroids, e.g. estradiol or testosterone<br>• Cross reactivity with digoxin: not known<br>• Conjugate does not bind to itself at all<br>• Normally one molecule of the conjugate binds to one molecule digoxigenin, although ther are two possible binding sites for digoxigenin<br>• Nonspecific binding to RNA is not expected<br><br>More info: www.sigmaaldrich.com/deepweb/assets/sigmaaldrich/product/documents/329/822/11093274910.pdf<br><br>Here is a list of >900 citations in the manufacturer's website:<br>www.sigmaaldrich.com/US/en...search

Animals and other research organisms

Policy information about studies involving animals; ARRIVE guidelines recommended for reporting animal research, and Sex and Gender in Research

Laboratory animals	All animals used in this study were of Mus musculus species and FVB/NCrl strain at ages E10.5, E11.5, E13.5, E18.5, and P0. We followed standard housing specifications as outlined in IACUC Policy on Animal Housing & Environmental Enrichment: 12-hour light-dark cycle, 20-26C, humidity between 30-70%.
Wild animals	Study did not use wild animals.
Reporting on sex	Gender was not identified during embryo collections; it is assumed that the groups contained approximately equal numbers of male and female mice
Field-collected samples	Study did not use field-collected samples.
Ethics oversight	All animal work was reviewed and approved by the Lawrence Berkeley National Laboratory Animal Welfare and Research Committee and the University California Irvine Laboratory Animal Resources (ULAR).

Note that full information on the approval of the study protocol must also be provided in the manuscript.

Plants

Seed stocks	N/A
Novel plant genotypes	N/A
Authentication	N/A
